# Investigating the relationship between the immune response and the severity of COVID-19: a large-cohort retrospective study

**DOI:** 10.3389/fimmu.2024.1452638

**Published:** 2025-01-08

**Authors:** Riccardo Giuseppe Margiotta, Emanuela Sozio, Fabio Del Ben, Antonio Paolo Beltrami, Daniela Cesselli, Marco Comar, Alessandra Devito, Martina Fabris, Francesco Curcio, Carlo Tascini, Guido Sanguinetti

**Affiliations:** ^1^ Physics Department, International School for Advanced Studies (SISSA), Trieste, Italy; ^2^ Infectious Disease Unit, Azienda Sanitaria Universitaria Friuli Centrale (ASU FC), Udine, Italy; ^3^ Department of Medicine (DMED), University of Udine, Udine, Italy; ^4^ Department of Laboratory Medicine, ASU FC, Udine, Italy

**Keywords:** COVID-19, cytokines, immunology, inflammation, flow cytometry, immune system, predictive modeling

## Abstract

The COVID-19 pandemic has left an indelible mark globally, presenting numerous challenges to public health. This crisis, while disruptive and impactful, has provided a unique opportunity to gather precious clinical data extensively. In this observational, case-control study, we utilized data collected at the Azienda Sanitaria Universitaria Friuli Centrale, Italy, to comprehensively characterize the immuno-inflammatory features in COVID-19 patients. Specifically, we employed multicolor flow cytometry, cytokine assays, and inflammatory biomarkers to elucidate the interplay between the infectious agent and the host’s immune status. We characterized immuno-inflammatory profiles within the first 72 hours of hospital admission, stratified by age, disease severity, and time elapsed since symptom onset. Our findings indicate that patients admitted to the hospital shortly after symptom onset exhibit a distinct pattern compared to those who arrive later, characterized by a more active immune response and heightened cytokine activity, but lower markers of tissue damage. We used univariate and multivariate logistic regression models to identify informative markers for outcome severity. Predictors incorporating the immuno-inflammatory features significantly outperformed standard baselines, identifying up to 59% of patients with positive outcomes while maintaining a false omission rate as low as 4%. Overall, our study sheds light on the immuno-inflammatory aspects observed in COVID-19 patients prior to vaccination, providing insights for guiding the clinical management of first-time infections by a novel virus.

## 1 Introduction

The COVID-19 pandemic had a devastating impact worldwide, causing significant morbidity and mortality. The strain on healthcare systems has been particularly pronounced, with hospitals reaching their capacity, especially for intensive care. Notably, the virus has disproportionately affected certain demographic groups, revealing stark disparities in health outcomes (1). The reasons for these disparities are multifaceted, encompassing socioeconomic and genetic factors, pre-existing health conditions, and, potentially, differences in immune responses. While our comprehension of the complex interplay between SARS-CoV-2 and the human immune system has improved over the years, the picture is still incomplete. During the early phase of the pandemic, it became evident that the severity of COVID-19 was influenced by the virus itself as well as the host’s immune response. An overactive or dysregulated response in some individuals led to severe disease and even death, while others exhibited a more controlled response leading to a better outcome (2, 3). The mechanisms governing these varied responses remain incompletely understood, emphasizing the need for in-depth studies on the interaction between the virus and the immune system.

Understanding the immune-inflammatory response to COVID-19 is also crucial for gaining insights into related diseases. One important example is *sepsis*, a severe condition that can be caused by bacteria, fungi, or viruses, which currently lacks a specific treatment (4). COVID-19 hospitalized patients should intrinsically be regarded as septic: the majority of critically ill patients (78%) met Sepsis-3 criteria for septic shock with acute respiratory distress syndrome (ARDS) as the most frequent organ dysfunction (88%) (5, 6). Recent advances in understanding sepsis have led to the belief that the majority of sepsis-related deaths are not caused by the initial hyperinflammatory state, but rather by the suppression of the immune system, known as sepsis-induced immunoparalysis (7). One of the mainstays of treatment for severely ill COVID-19 patients on supplemental oxygen has been glucocorticoids, which are anti-inflammatory and immunosuppressive drugs (8). However, for some COVID-19 and septic patients, immunostimulation rather than immunosuppression can be a more appropriate approach: understanding the underlying mechanisms of disease progression is essential to prevent inappropriate treatments. Furthermore, a deeper understanding of the immune response to SARS-CoV-2 could aid in developing predictive tools to identify individuals at risk of severe outcomes. This would be extremely important for prioritizing hospitalizations and allocating healthcare resources. Clinical scores have been utilized as a method for predicting outcomes and stratifying risks, such as the 4C Mortality Score, and they have shown promising results (9, 10).

Throughout the pandemic, our hospital, the *Ospedale Santa Maria della Misericordia* of Udine (Italy), has witnessed a large number of hospitalizations among COVID-19 patients, presenting an unprecedented opportunity to accumulate extensive information about a single disease. We therefore used the collected clinical data to create a comprehensive retrospective database, the MANDI registry (“MAnagement coroNavirus Disease In hospital registry” – authorization of DG, decree n. 957, 10/09/2021). This database has allowed us to delve into the study of immune status and identify mid-regional pro-adrenomedullin (proADM) as an effective biomarker for predicting outcomes, in association with lactate dehydrogenase (LDH) and C-reactive protein (CRP) (11, 12). Additionally, we have investigated the role of cytokines in the setting of COVID-19-related pericarditis (13) and employed machine learning techniques to develop predictive tools while deepening our understanding of cytokines and serum proteomics (14, 15). Building upon these achievements, in the present paper we aim to provide a more comprehensive study of the immuno-inflammatory profiles of COVID-19 patients. Our focus is on analyzing the distribution of monocyte and lymphocyte populations observed during the infection, utilizing immunological data obtained by flow cytometry. Additionally, our analysis includes serological biomarkers (CRP, proADM, and LDH), as well as cytokines, thus covering different aspects of the host’s immuno-inflammatory response. We believe that a better comprehension of the pathophysiology of COVID-19 could provide insights into the broader management of sepsis. The contribution of this study is twofold. Firstly, we present a detailed phenotypic characterization of COVID-19 patients, with an emphasis on viral etiology, shedding light on the specific immune cell profiles associated with the infection. Secondly, we conduct a predictive analysis to identify the most informative biomarkers for predicting patient outcomes, using several clinical scores as baselines for comparison.

The rest of the manuscript is organized as follows: in Sec. 2, we characterize the patients’ immune-inflammatory response through descriptive statistics. Sec. 3 presents our analysis aimed at predicting patient outcomes. Given the multifaceted nature of our analyses across different data types, we discuss the results within their respective sections as they are presented. Additionally, we provide a comprehensive summary of the main findings and a broader discussion in Sec. 4. Finally, a detailed description of the materials and methods employed in our study is provided in Sec. 5.

## 2 Immuno-inflammatory profiles of COVID-19 patients

The data collection process was conducted between March 2020 and April 2021, covering the first three waves of the pandemic. The dataset consists of approximately 900 records and includes a range of variables collected at hospital admission. These variables encompass demographic details, individual comorbidities, monocyte and lymphocyte counts, and, for smaller subsets of patients, cytokines and serological biomarkers measurements. Importantly, none of the patients had been vaccinated against SARS-CoV-2 at the time of data collection. Below is a complete list of all the immuno-inflammatory features considered in this study, along with the abbreviations used throughout the manuscript:

- flow cytometry (FC set): counts of white blood cells (WBC), monocytes (Mono), lymphocytes (Lymph), T and B cells with corresponding CD positivity (CD3, CD4, CD8, CD19), natural killer cells (NK), recent thymic emigrants (RTE), the percentage of monocytes, CD4, and CD8 positive cells expressing HLA-DR ( $\text{H}\text{L}{\text{A}}_{\%\text{M}\text{o}\text{n}\text{o}}^{+}$ , $\text{H}\text{L}{\text{A}}_{\%\text{C}\text{D}4}^{+}$ , $\text{H}\text{L}{\text{A}}_{\%\text{C}\text{D}8}^{+}$ , respectively), the percentage of RTE-CD4 cells $\left(\text{R}\text{T}{\text{E}}_{\%\text{C}\text{D}4}\right)$ , and the monocytes HLA-DR mean intensity fluorescence $\left(\text{H}\text{L}{\text{A}}^{+}\text{I}{\text{F}}_{\text{M}\text{o}\text{n}\text{o}}\right)$ .
- cytokines (CK set): interleukin 10 (IL10), IL1-*β* (IL1B), sIL2R-*α*/sCD25 (IL2R), interleukin 6 (IL6), interleukin 8 (IL8), chemokine IP10/CXCL10 (IP10), and interferon-*γ* (INF-*γ*).
- biomarkers (BM set): mid-regional pro-adrenomedullin (proADM), a marker of endothelial response to inflammation and tissue damage, lactate dehydrogenase (LDH), a marker of cell proliferation and/or damage, and C-reactive protein (CRP), a marker of inflammation.

We also collected flow cytometry and demographic data from approximately 370 asymptomatic outpatients, referred to as the *control set* in our analysis. Importantly, the control set has statistical similarities with a smaller cohort of healthy individuals (around 90 cases), as shown in **Supplementary Figure 2**. For hospitalized patients, we collected information on outcomes, including survival status, as well as the treatments they received. Disease severity was assessed using a 4-point ordinal scale, based on the World Health Organization’s guidance (16), which we refer to as the WHO scale.

Depending on data availability, we also applied the Charlson Comorbidity Index and four clinical indices as baseline scores to evaluate patients’ conditions at hospital admission, as detailed below:

- CCI [Charlson Comorbidity Index (17)]: though not a conventional score, it is used to predict the ten-year mortality for patients with various comorbid conditions. Each condition is assigned a score of 1, 2, 3, or 6, depending on the associated risk of mortality.
- SOFA [Sequential Organ Failure Assessment (18)]: assesses six organ systems (respiratory, cardiovascular, hepatic, coagulation, renal, and neurological). It is used for mortality prediction in intensive care unit patients (19) and for the diagnosis of sepsis (18).
- NEWS [National Early Warning Score (20)]: evaluates the severity of a patient’s illness and indicates the need for critical care intervention (21). It assesses respiration rate, oxygen saturation, systolic blood pressure, pulse rate, level of consciousness, and temperature.
- qCSI [Quick COVID-19 Severity Index (22)]: based on respiratory rate, pulse oximetry, and speech evaluation, it predicts the 24-hour risk of critical respiratory illness in COVID-19 patients.
- 4C [Coronavirus Clinical Characterisation Consortium mortality score (23)]: incorporates age, sex, number of comorbidities, respiratory rate, peripheral oxygen saturation, level of consciousness, urea level, and CRP to predict the in-hospital mortality of patients admitted with COVID-19 (24, 25).

In the following sections, we provide a detailed analysis of the flow cytometry, cytokines, and biomarkers variables, focusing on their relationships with illness severity (WHO scale), patient age, and the number of days between symptom onset and hospitalization (Δt_ons_). It is important to note that Δt_ons_ is an anamnestic variable with certain limitations, as it depends on the patient’s ability to recognize symptoms and accurately recall when they first appeared. For our analysis, we included only patients aged 30 to 100 years and with Δt_ons_ ranging 0 to 30 days. Patients with a CCI greater than 6 were excluded to minimize the influence of confounding factors on the outcomes. The resulting records, grouped based on the availability of data for the three feature sets (FC, CK, BM), show homogeneity in characteristics, as summarized in **Table 1**, with the exception of sample size, which is largest for the FC set. However, it should be noted that outpatients tend to be younger and include a higher proportion of females. For further details on the data collection process and preprocessing, we refer to Sec. 5.

**Table 1:** Demographics and clinical characteristics, for all records (FC set), and subsets of the FC set with measurements of cytokines (CK set) and serological biomarkers (BM set).

outpatients						
set	N	sex (female)	age [Q_2_(Q_1_-Q_3_)]	Δt_ons_ [Q_2_(Q_1_-Q_3_)]	CCI *<* 2	NANs
FC	367	58.3%	51 (36-61)	–	–	22.1%
inpatients						
set	N	sex (female)	age [Q_2_(Q_1_-Q_3_)]	Δt_ons_ [Q_2_(Q_1_-Q_3_)]	CCI *<* 2	NANs
FC	788	33.9%	68 (58-76)	9 (7-12)	20.3%	3.8%
CK	297	34.3%	67 (56-77)	9 (7-12)	22.7%	0.9%
BM	539	35.1%	68 (58-76)	9 (7-12)	20.5%	6.9%
set	SOFA > 1	NEWS > 4	qCSI > 6	4C > 8	WHO > 2	OTI+death
FC	63.0%	28.3%	2.0%	48.7%	63.2%	24.1%
CK	61.4%	22.8%	0.7%	46.5%	58.0%	23.6%
BM	60.5%	24.0%	1.7%	51.0%	57.9%	20.8%

Only records with less than 50% missing data (NANs) were included in each set. Dichotomous variables are presented as percentages of available data. Numerical variables are described by the median (Q_2_), first (Q_1_), and third quartile (Q_3_). The cutoff of the scores are those identified in the literature as pathological. Dashes indicate missing information.

### 2.1 Relationship between immune response and disease severity

**Figure 1:**
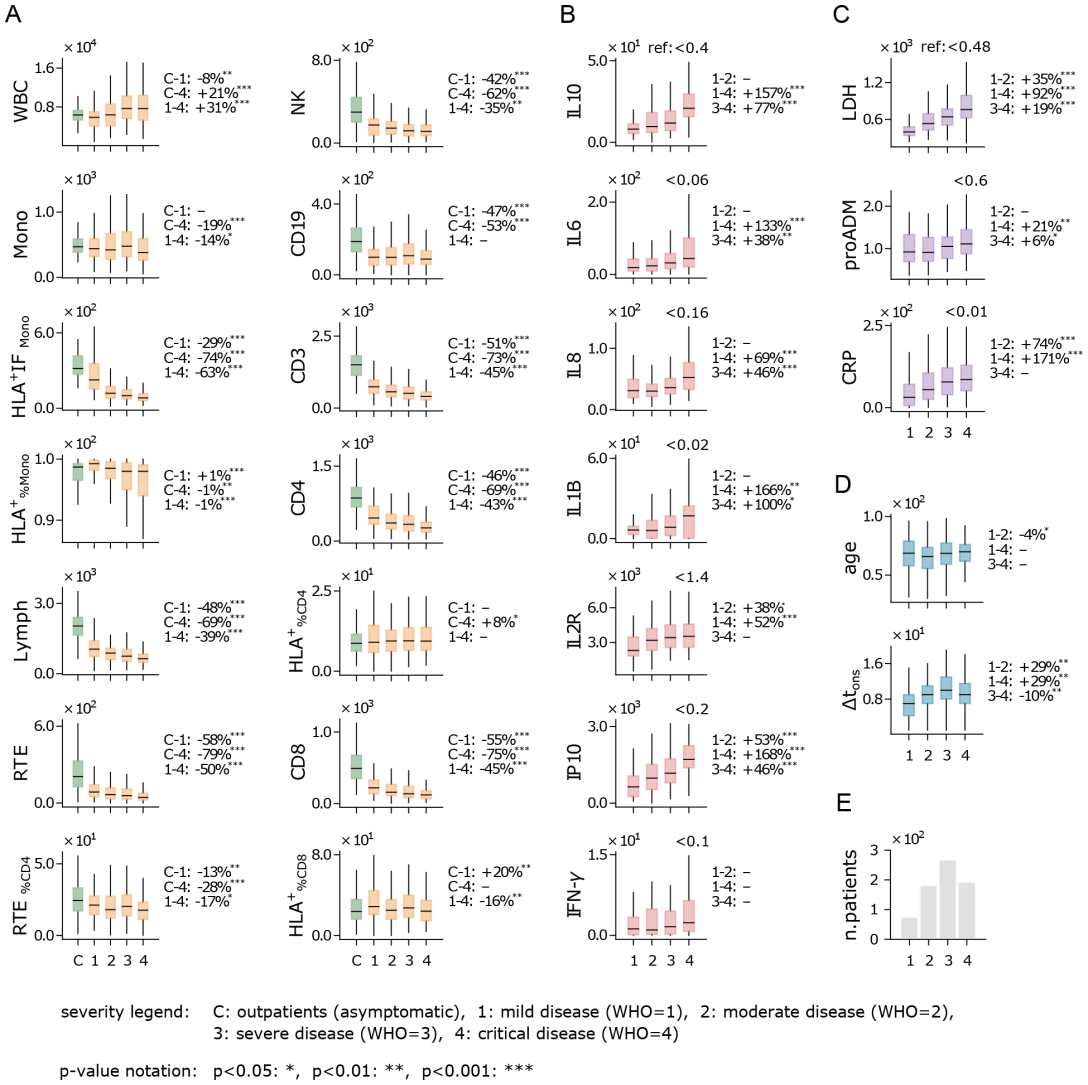
Immune response and disease severity. Boxplot of flow cytometry variables **(A)**, cytokines **(B)**, biomarkers **(C)**, age and Δt_ons_ **(D)**, for patients stratified by the highest disease severity level reached during hospitalization (see legend). Measurements were taken at hospital admission. Boxes span first to third quartiles, with the horizontal bar representing the median. The percentage change of the median between severity levels is shown on the right of each plot (example: the WBC median is 8% lower in patients with mild disease compared to outpatients). Reference values for healthy individuals are shown at the top-right of plots with no control cohort. **(E)** Number of hospitalized patients across disease severity levels (WHO scale). Measurement units: white blood cells (WBC) and related subpopulations (Lymph, Mono, CD3, CD4, CD8, NK, CD19, RTE): U/*µ*L; cytokines (IL10, IL6, IL8, IL1B, IL2R, IP10, INF-*γ*): pg/mL; lactate dehydrogenas (LDH): U/L; mid-regional pro-adrenomedullin (proADM): nmol/L; C-reactive protein (CRP): mg/L.

We investigated how flow cytometry measurements, cytokine levels, and biomarker concentrations varied with the severity of disease during hospitalization; the results are shown in **Figure 1**. Disease severity was categorized using the WHO scale, which defines severity as mild (1), moderate (2), severe (3), and critical (4); further details are provided in Sec. 5. **Figure 1A** illustrates the flow cytometry features, where we observe a non-monotonic trend in white blood cell (WBC) counts: WBC are lower in inpatients with mild disease compared to outpatients (control) and increase as disease severity worsens among inpatients. Interestingly, the average number of monocytes (Mono) remains relatively stable across WHO severity levels, while their activation (HLA^+^IF_Mono_) decreases sharply as severity increases. Conversely, a marked reduction is seen in lymphocyte counts, both in aggregate (Lymph) and in specific subpopulations such as CD4, CD8, and NK cells. Given that monocyte counts remain stable and lymphocyte counts decrease, the observed increase in WBCs with disease severity is most likely driven by granulocyte populations, such as neutrophils. Although granulocyte counts were not directly measured in this study, it is well-established that neutrophil counts rise significantly in severe inflammatory responses and infections, including severe COVID-19 cases, contributing to the overall increase in WBCs as disease severity progresses (26). Notably, CD19 counts are lower in inpatients compared to the outpatient control set, although differences across WHO severity levels are minimal and not significant.

Cytokines show a consistent overexpression relative to reference values (**Figure 1B**), with levels rising as WHO severity increases. This trend is particularly evident for IP10, a pro-inflammatory chemokine that recruits immune cells to infection sites, and IL10, an anti-inflammatory cytokine that helps regulate immune responses and mitigate excessive tissue damage. A sharp increase in IL1B, a key cytokine in infection response, is also noted, along with IL6, a critical pro-inflammatory mediator that induces most acute phase reactants and is closely linked to CRP (27, 28). While recent studies have shown that patients with severe COVID-19 tend to have lower levels of interferon-gamma (IFN-*γ*) compared to those with milder forms of the disease (29, 30), in our analysis IFN-*γ* shows a moderate (non-significant) increase with disease severity. Overall, the rising cytokine levels are consistent with the well-documented “cytokine storm” phenomenon observed in COVID-19 patients, characterized by elevated levels of both pro-inflammatory signals (IL6, IL8, and IP10) and immunomodulatory signals (IL10 and IL2R) (31, 32).

Similarly to cytokine levels, all analyzed biomarkers display elevated concentrations and an upward trend with increasing WHO severity levels (**Figure 1C**). This trend is particularly pronounced for lactate dehydrogenase (LDH) and C-reactive protein (CRP), both of which are widely recognized as markers of systemic inflammation. The strong correlation between these biomarkers and WHO severity levels suggests greater systemic involvement in more severe cases of COVID-19. Notably, pro-adrenomedullin (proADM), despite being a well-established predictor of patient outcomes in previous studies (11, 33), exhibits only moderate variation across the WHO scale in our dataset.

Interestingly, the age distribution of patients shows no significant variation when stratified by WHO severity levels (**Figure 1D**). This suggests that within the studied cohort, age is not a strong determinant of the risk of developing severe COVID-19 symptoms. Although this appears to contradict earlier findings that linked older age with increased disease severity (34), it is important to clarify that those studies referred to age-related risks in terms of severe outcomes – such as in-hospital mortality, case fatality, and hospitalization – rather than the severity of the disease symptoms themselves. Additionally, **Figure 1D** shows that patients who develop milder forms of COVID-19 tend to arrive at the hospital earlier (i.e. have a shorter Δt_ons_) compared to those who progress to more severe illness. Finally, it is important to note that the majority of hospitalized patients reached WHO level 3 (**Figure 1E**), which represents a critical point between patients with mild to moderate symptoms and those with critical conditions. This WHO level includes both patients with positive outcomes (recovery) and those with negative outcomes (death or worsening), highlighting the complexity of assessing disease severity based solely on clinical presentation. This underscores the challenge of predicting outcomes at this stage, as WHO level 3 encompasses a wide range of disease trajectories.

### 2.2 The impact of age on the immune response

**Figure 2:**
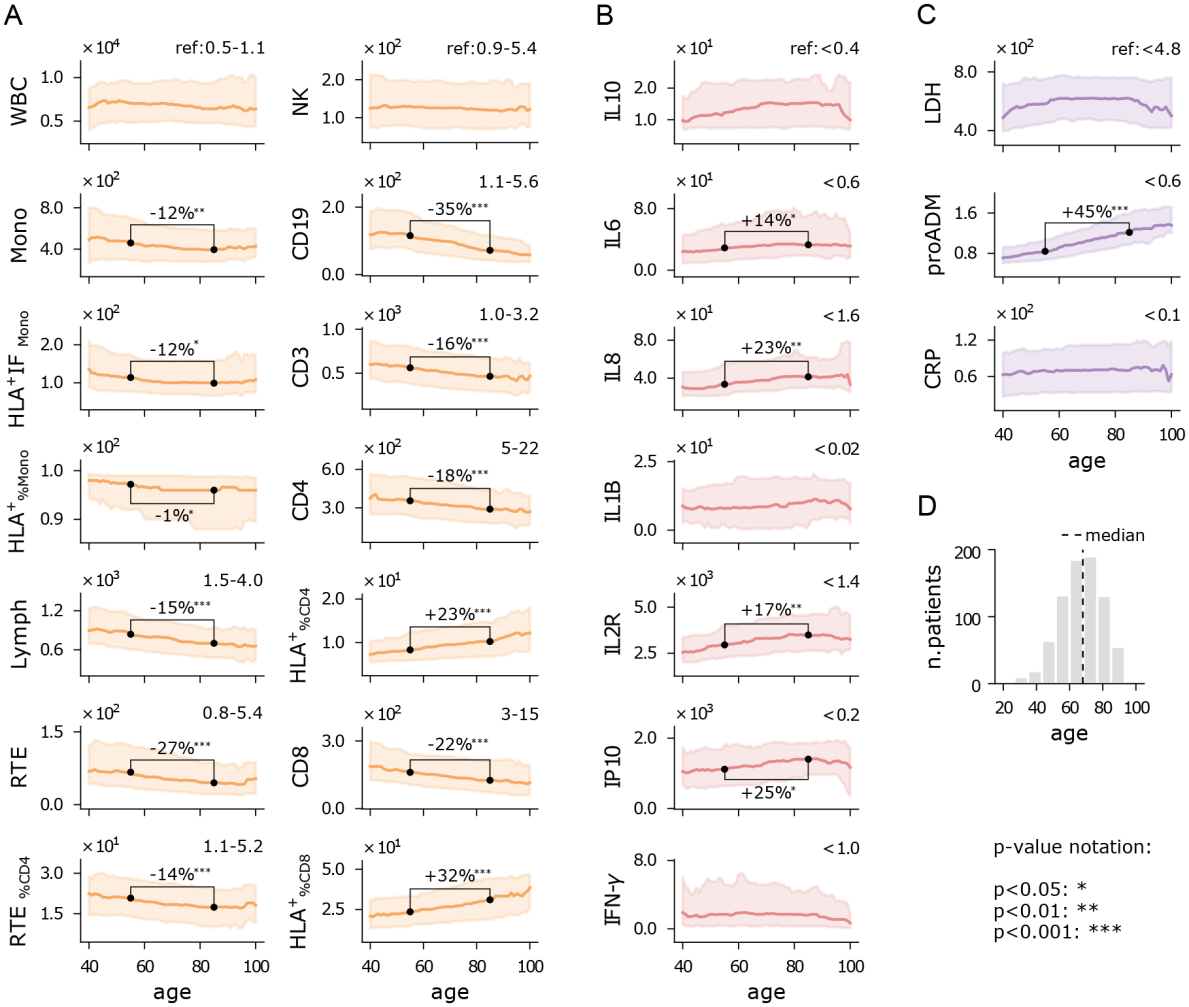
Immuno-inflammatory features vs age. Running median with a 15-year half-window of flow cytometry variables **(A)**, cytokines **(B)**, and biomarkers **(C)** measured at hospital admission. Shaded areas represent the first to third running quartiles. Significant changes in distribution between ages 55 and 85 are expressed as the percentage increase or decrease of the median. Reference values for healthy individuals are shown in the top-right corner of each plot, where available. **(D)** Age histogram of hospitalized patients. Measurement units: white blood cells (WBC) and related subpopulations (Lymph, Mono, CD3, CD4, CD8, NK, CD19, RTE): U/*µ*L; cytokines (IL10, IL6, IL8, IL1B, IL2R, IP10, INF-*γ*): pg/mL; lactate dehydrogenas (LDH): U/L; mid-regional pro-adrenomedullin (proADM): nmol/L; C-reactive protein (CRP): mg/L.

Increased age has been reported as a significant factor in severe COVID-19 outcomes across several studies (34). Indeed, aging is frequently associated with immunosenescence, a process characterized by a decline in immune cell function and diversity, which weakens a person’s ability to mount effective immune responses (35). In this study, we aimed to identify the specific aspects of the immune system most affected by the SARS−CoV−2 viral infection, while also considering the role of immunosenescence. To this end, we analyzed the rolling median of each immune feature over a 15-year half-window, with the results presented in **Figure 2**. Additionally, we conducted a quantitative comparison between patients aged 40 to 70 years and those aged 70 to 100 years, as 70 years aligns approximately with the median age of hospitalized patients (**Figure 2D**). A direct comparison between the cohorts of inpatients and outpatients is provided in **Supplementary Figure 1**.

**Figures 2A** illustrates the flow cytometry features. We observe that the median counts of white blood cells (WBC) and natural killer cells (NK) remain relatively stable across the age spectrum, with values near the lower end of the reference range. Monocytes show a slight decline with age, both in absolute numbers (Mono) and in functionality (HLA^+^IF_Mono_). In contrast, lymphocytes (Lymph) display a pronounced decrease with age, falling below the reference range, particularly for recent thymic emigrants (RTE) and CD19 cells. This reduction suggests a trend towards granulocytosis with increasing age. Interestingly, the percentage of CD4 and CD8 cells expressing HLA-DR increases with age, indicating a reduction of inactive lymphocytes, though overall lymphocyte activity may still be diminished in older patients. Further analysis of CD4 and CD8 intensity fluorescence (HLA^+^IF) would be required to confirm this observation. The relative increase of $\text{H}\text{L}{\text{A}}_{\%\text{C}\text{D}4}^{+}$ and $\text{H}\text{L}{\text{A}}_{\%\text{C}\text{D}8}^{+}$ , alongside a reduction in CD19, could indicate a shift towards a more cellular-based immune response rather than a humoral one. These trends are also seen in the younger cohort of outpatients, suggesting that immunosenescence may occur independently of disease severity (**Supplementary Figure 1**). However, outpatients exhibit higher lymphocyte counts, with a less pronounced decline with age, particularly in RTE and CD19 cells. Additionally, their monocyte counts do not show significant age-related changes. Remarkably, unlike inpatients, outpatients do not experience a reduction in CD4 and CD8 cell counts, though they still show an increase in $\text{H}\text{L}{\text{A}}_{\%\text{C}\text{D}4}^{+}$ and $\text{H}\text{L}{\text{A}}_{\%\text{C}\text{D}8}^{+}$ with age, similar to inpatients. Overall, patients who did not require hospitalization demonstrated a stronger and more consistent immune response across age groups compared to those who were hospitalized.

Cytokine levels remain high above reference values across all ages (**Figure 2B**), reflecting the persistent dysregulated inflammatory state in COVID-19 patients. Most cytokines exhibit a moderate upward trend with age, particularly IL8 and IL2R, likely due to age-related chronic low-grade inflammation, which manifests as higher baseline cytokine levels (36).

All the inflammatory biomarkers show median values above the reference range in patients of all ages (**Figure 2C**). However, pro-adrenomedullin (proADM) is the only biomarker that exhibits a strong positive correlation with age. Other studies have highlighted the interaction between proADM and age in predicting clinical outcomes, such as cardiovascular events (37), or severe COVID-19 outcomes (11), but the specific nature of this interaction remains an open question.

### 2.3 Temporal patterns of the immune response

COVID-19 patients typically presented to the hospital approximately 10 days after symptom onset, a time frame we refer to as Δt_ons_, although this duration varied from 1 day to over a month. This variability reflects differences in disease progression dynamics. A shorter Δt_ons_ suggests a rapid, acute onset that required immediate hospitalization, while larger values suggest a more gradual disease course, where hospitalization became necessary only at a later stage.

To explore the relationship between the immune response and Δt_ons_, we analyzed the rolling median of each feature using a 5-day half-window across the Δt_ons_ range, providing insights into how these feature change over time. We also compared the distributions of immune features for two subgroups: those hospitalized within 1 to 10 days and those within 11 to 20 days. It is important to note that our data does not track individual patients over time; rather, it provides a snapshot of how each variable correlated with Δt_ons_, instead of depicting personal disease trajectories.

**Figure 3:**
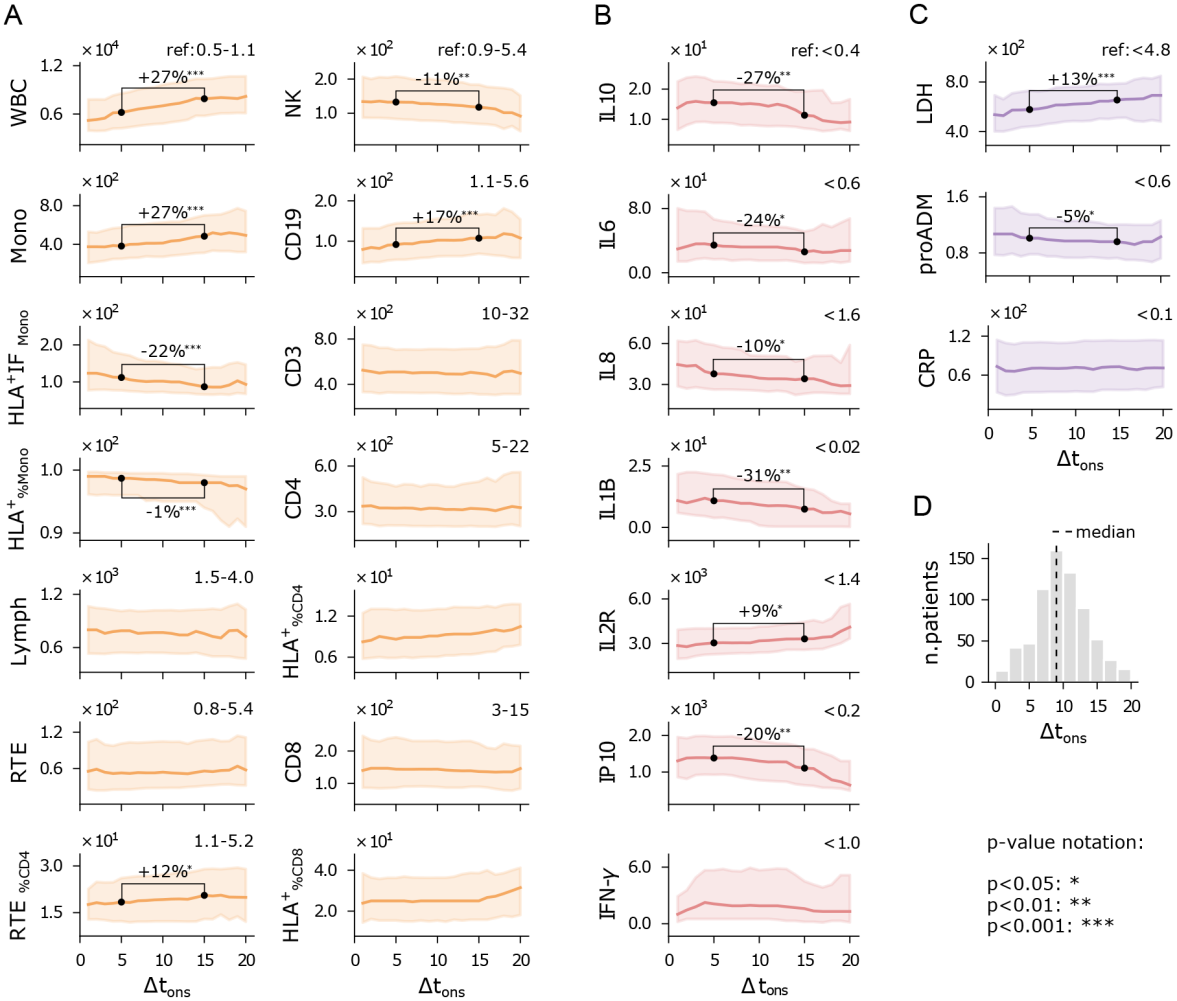
Immuno-inflammatory features vs days from symptom onset to hospitalization (Δt_ons_). Running median with a 5-day half-window of flow cytometry variables **(A)**, cytokines **(B)**, and biomarkers **(C)** measured at hospital admission. Shaded areas represent the first to third running quartiles. Significant changes in distribution between Δt_ons_ of 5 and 15 days are expressed as the percentage increase or decrease of the median. Reference values for healthy individuals are shown in the top-right corner of each plot, where available. **(D)** Δt_ons_ histogram of the hospitalized patients. Measurement units: white blood cells (WBC) and related subpopulations (Lymph, Mono, CD3, CD4, CD8, NK, CD19, RTE): U/*µ*L; cytokines (IL10, IL6, IL8, IL1B, IL2R, IP10, INF-*γ*): pg/mL; lactate dehydrogenas (LDH): U/L; mid-regional pro-adrenomedullin (proADM): nmol/L; C-reactive protein (CRP): mg/L.

Despite the inherent variability of Δt_ons_ as a self-reported, anamnestic variable, clear trends emerge (**Figure 3**). Patients with higher Δt_ons_ show elevated white blood cell (WBC) counts, monocytes, and CD19 cells, but reduced monocyte activation (HLA^+^IF_Mono_) and natural killer (NK) cells (**Figure 3A**). Meanwhile, T cell numbers remain relatively stable across the Δt_ons_ range. The rise in WBC counts appears to be driven by increased monocyte and granulocyte numbers, potentially compensating for the declining functionality of monocytes. Although NK and CD19 cell levels are reduced, we observe a typical immune trajectory over time: as Δt_ons_ increases, there is a shift from innate response (NK) to adaptive immune activation (CD19).

Cytokine expression levels generally show a declining trend with increasing Δt_ons_ (**Figure 3B**), particularly IL10, IL1B, and IP10. Higher cytokine levels in patients with shorter Δt_ons_ point to a more intense hyperinflammatory state early on, while patients with longer Δt_ons_ still exhibit elevated cytokines, though at reduced levels, suggesting a waning inflammatory response. This trend may reflect the natural resolution of inflammation over time, but longitudinal measurements would be needed to confirm this interpretation. Finally, lactate dehydrogenase (LDH) levels indicate that patients who arrive earlier at the hospital experience less systemic damage (**Figure 3C**). Similarly, proADM levels are higher in patients with shorter Δt_ons_, mirroring the behavior of pro-inflammatory cytokines.

Overall, these findings suggest that inflammation and cytokine levels are typically elevated when symptoms first appear, and tend to decrease in patients with slower disease progression who arrive later at the hospital. This indicates that patients hospitalized shortly after symptom onset often present with a more acute inflammatory response, while those admitted later exhibit more advanced immune responses and greater tissue damage. It is important to note that this is a cross-sectional analysis, and longitudinal data would be required to fully understand the dynamics of disease progression. Nonetheless, these results underscore the importance of accounting for immune response dynamics when evaluating a patient’s clinical condition.

## 3 Outcome prediction analysis

**Figure 4:**
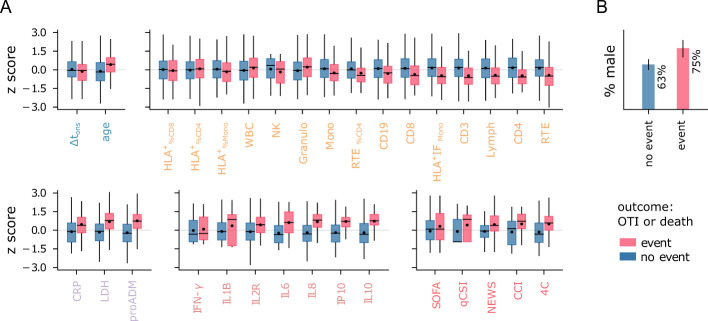
*Features vs outcome.* **(A)** Distribution of normalized features stratified by patient outcome. Boxes span first to third quartiles. The horizontal bar and black dot represent the median and mean, respectively. **(B)** Percentage of male patients in each outcome group. The legend refers to both panels.

The ability to mount an effective response to the infection significantly impacts patients’ prognosis. As discussed in Sec. 2.1, reduced levels of lymphocytes and NK cells are typically associated with more severe disease progression, as reflected in the WHO severity scale. However, while the WHO scale measures the severity of illness, it does not provide direct information about patient outcomes, such as mortality. Features strongly linked to disease severity may not be equally relevant for predicting patient outcomes, making them less useful for clinical decision-making. For instance, while pro-adrenomedullin shows only a weak correlation with WHO severity levels (see **Figure 1E**), it has been identified as a powerful predictor of survival rates (11, 12). To identify the immune-inflammatory markers with the highest predictive power for clinical outcomes, we focused on classifying patients based on the combined outcome of death and/or orotracheal intubation, referred to as the *death+OTI* outcome. A visual representation of the immune-inflammatory features and clinical scores stratified by the death+OTI outcome is provided in **Figure 4**. This joint outcome has two advantages over mortality alone: it increases the number of cases in the minority class (patients with negative outcomes) and reduces bias introduced by prioritization for invasive and life-saving interventions.

We employed logistic regression (LR) models for our analysis, as preliminary tests with non-linear classifiers such as support vector machines and random forests did not show significant improvements. Two aspects of the LR models were evaluated: overall predictive performance, measured by the area under the ROC curve (AUC), and the ability to accurately identify low-risk patients. Specifically, we aimed to detect the largest number of patients who did not experience negative outcomes while keeping the rate of false negatives below a predetermined low threshold. This second evaluation criterion is particularly important for establishing priority protocols during critical situations, such as pandemic outbreaks. We set a minimum negative predictive value (NPV) requirement of 0.97 for classifiers on the training set, with NPV values above 0.95 considered acceptable for the test set. Although these thresholds are illustrative, it is important to note that test NPV is typically lower than a training NPV set to a very high value, and the required threshold must be adjusted accordingly. Detailed information on data preprocessing and model specifications is provided in Sec. 5.

### 3.1 Cytokine-based univariate models outperform baseline indices

**Figure 5:**
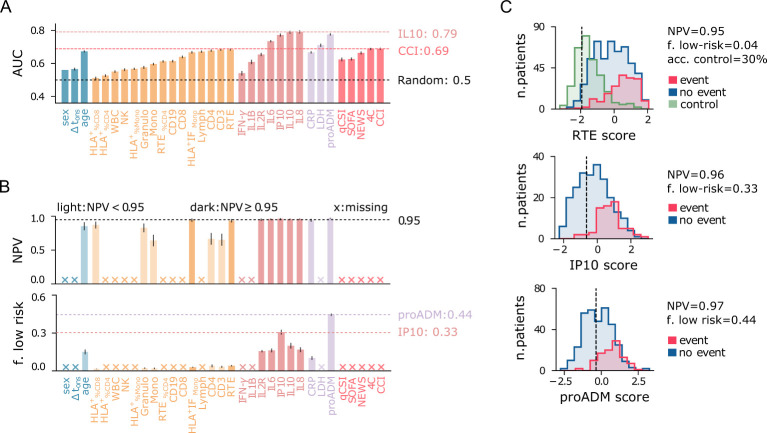
Performance of univariate predictive models for the combined outcome event of death and/or orotracheal intubation. **(A)** Area under the ROC curve (AUC) for each predictor variable shown on the x-axis. **(B)** Negative predictive value (NPV) and fraction of low-risk patients correctly identified (specificity) for predictor variables on the x-axis (as in panel A), conditioned on train NPV ≥ 0.97. Crosses on the x-axis denote models that do not meet this criterion. Light-colored bars indicate models with test NPV < 0.95. Error bars represent 95% confidence intervals. **(C)** Logistic regression score histograms for patients with positive (blue) and negative (red) outcomes, and for outpatients (green, control), highlighting the best predictors among flow cytometry features, cytokines, and biomarkers. The vertical line indicates the cutoff threshold for the minimum NPV requirement.

Firstly, we evaluated the predictive power of individual features using univariate LR models, excluding missing data from the analysis. The results are displayed in **Figure 5**. Among the clinical indices, the Charlson Comorbidity Index (CCI) and the 4C score show the highest AUC values (**Figure 5A**). For further comparisons, we use the 4C score as the best baseline predictor and its associated AUC as a reference (AUC_ref_ = 0.69). Remarkably, several flow cytometry features, cytokines, and biomarkers match or outperform this baseline. Among the flow cytometry features, lymphocytes (RTE, CD3, CD4, Lymph) and monocyte activation (HLA^+^ IF_Mono_) emerge as the strongest predictors, with RTE and CD3 achieving AUC values equal to AUC_ref_. Interestingly, granulocytes tend to be more highly expressed in patients with negative outcomes (**Figure 4**), contrasting with the lower levels of monocytes and lymphocytes. As a result, the total WBC count has limited predictive power compared to these three subpopulations. It is important to note that granulocyte counts were not measured directly in this study; instead, they are estimated by subtracting lymphocyte and monocyte counts from the total WBC count. The reference AUC_ref_ value is significantly exceeded by mid-regional pro-adrenomedullin (proADM), which confirmed its strength as a predictor of clinical outcomes, and by several cytokines. IL8 and IL10 are the top performers, with the highest AUC values of all univariate LR models (AUC = 0.79). IL8 is a potent, proinflammatory chemokine that induces degranulation of neutrophils and adhesion of polymorphonuclear cells to the endothelium. It is released by various cell types in response to inflammation, including monocytes, macrophages, and neutrophils (38). This pro-inflammatory mechanism mediated by IL8 likely explains the increase in both granulocytes and IL8 levels observed in patients with poor outcomes. In contrast, IL10 plays an essential role in inducing an immunoregulatory phenotype in B cells, exerting significant anti-inflammatory and immunosuppressive effects (39).

Finally, we confirm that age is a relatively good predictor, consistent with previous reports (40, 41), though its AUC is slightly below AUC_ref_. In contrast, sex and Δt_ons_ show significantly lower predictive power compared to the best baseline predictor. To summarize, poor outcomes are strongly associated with high cytokine expression, increased endothelial dysfunction (high proADM), cell injury (high LDH), and immune deficiency (IL10 overexpression, reduced lymphocytes, monocytes, and monocyte activation).

Identifying low-risk patients with high confidence using only one feature at a time poses a significant challenge, and none of the baseline clinical indices proves useful for this task (**Figure 5B**). Instead, the proADM-based model achieves a remarkable result, identifying 44% of negative cases with a false omission rate of only 3%. Low-risk patients can also be identified using cytokine expression levels, particularly IP10 (specificity = 0.31). Instead, among all flow cytometry features, only recent thymic emigrants (RTE) and monocyte activation (HLA^+^ IF_Mono_) fulfill the minimum NPV requirement on the test set, but with low specificity. The identified low-risk cutoff values are reported in **Table 2**. The score histogram of RTE, IP10, and proADM-based models are depicted in **Figure 5C**, where the vertical dashed line marks the threshold below which patients are considered at low risk. Remarkably, only 30% of outpatients would be correctly classified as low-risk with the RTE-based predictor. As we will see, this prediction improves dramatically when using multivariate models.

**Table 2:** Best univariate predictors for low-risk patient identification.

	RTE	IL10	IL2R (10^3^)	IL6	IL8	IP10 (10^2^)	proADM	CRP
**range**	3 - 346	2.3 - 85.7	0.8 - 11.0	2 - 504	9 - 255	1 - 36	0.45 - 3.34	1 - 269
**cutoff**	≥ 263	≤ 5.5	≤ 1.9	≤ 6.9	≤ 19	≤ 6.5	≤ **0.84**	≤ 9
**NPV**	0.93	0.96	0.95	0.96	0.96	0.96	0.97	0.94
**specificity**	0.04	0.20	0.16	0.16	0.17	0.31	**0.44**	0.10

Columns correspond to the features representing the best predictors. Rows show the observed range (1st–99th quantiles), the low-risk cutoff threshold, and the associated negative predictive value (NPV) and specificity for each feature. The cutoff and specificity of the best predictor (proADM) are highlighted in bold. Measurement units: RTE: U/*µ*L; cytokines (IL10, IL2R, IL6, IL8, IP10): pg/mL; proADM: nmol/L; CRP: mg/L.

### 3.2 Multivariate models improve identification of low-risk patients

The immuno-inflammatory features provide complementary information on the host’s response to the infection and, when combined, can lead to more accurate predictions. To this end, we employed multivariate LR models using the following feature sets.

- FC: T CD3 cells (CD3), percentage of RTE-CD4 cells (RTE_%CD4_), B CD19 cells (CD19), monocytes (Mono), monocyte HLA-DR intensity fluorescence (HLA^+^IF_Mono_), granulocytes (Granulo);
- CK: interleukins 1-*β* (IL1B), sIL2R-*α*/sCD25 (IL2R), 6 (IL6), 8 (IL8), 10 (IL10);
- BM: mid-reg. pro-adrenomedullin (proADM), lactate dehydrogenase (LDH), C-reactive protein (CRP).

We evaluated these sets individually and in combination with the demographic set (Dem), including sex, age, and Δt_ons_. Note that FC and CK are subsets of the sets introduced in Sec. 2, which we selected for their clinical relevance and predictive power while controlling for collinearity. Only cases with less than 50% missing data for each set were included in the multivariate analysis (**Supplementary Table 1**), and missing values were imputed using the KNN method.

**Figure 6:**
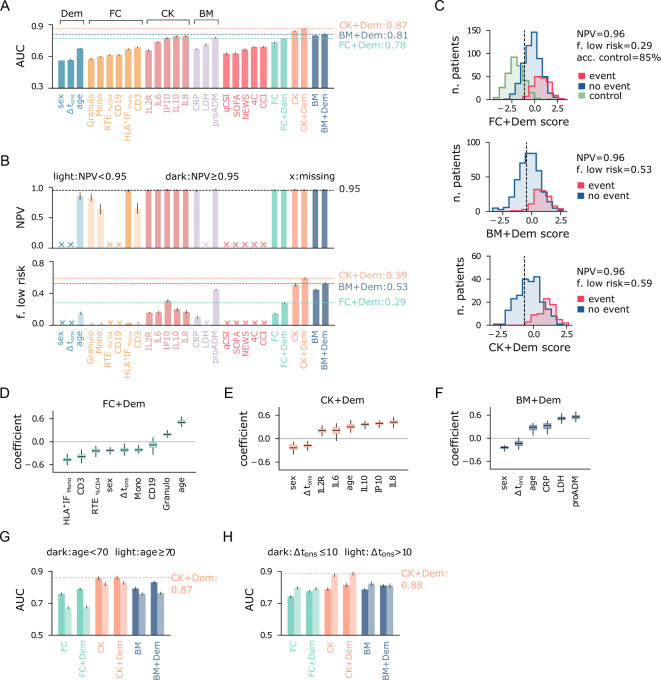
Performance of multivariate predictive models for the combined outcome event of death and/or orotracheal intubation. **(A)** Area under the ROC curve (AUC) of univariate and multivariate logistic regression models. Input variables are shown on the x-axis, and multivariate models are named according to the feature set used as input. **(B)** Negative predictive value (NPV) and fraction of low-risk patients correctly identified (specificity) by the predictive models, conditioned on train NPV ≥ 0.97. Crosses on the x-axis denote models that do not meet this criterion. Light-colored bars indicate models with test NPV < 0.95. **(C)** Score histograms of the best multivariate models employing the three main feature sets (FC, BM, CK), for patients with positive (blue) and negative (red) outcomes, and for outpatients (green, control). The vertical line indicates the cutoff threshold for the minimum NPV requirement. **(D)** Box plots of normalized coefficients from the FC+Dem model, **(E)** CK+Dem model, and **(F)** BM+Dem model. **(G)** Area under the ROC curve (AUC) of multivariate logistic regression models for patients stratified by age and **(H)** delay between symptom onset and hospitalization Δt_ons_. Error bars represent 95% confidence intervals.

The results of the multivariate prediction are summarized in **Figure 6**, where panel A shows the AUC of multivariate logistic regression models, and the corresponding univariate models using variables from the FC, CK, BM, and Dem sets. The best performances are again obtained using cytokines and serological biomarkers. In particular, the CK+Dem model achieves an AUC = 0.87, a significant improvement over the best baselines (AUC_ref_ = 0.69). This model also performs best in identifying low-risk patients, with specificity = 0.59 and NPV = 0.96 (**Figure 6B**), meaning that it correctly detects 59% of low-risk patients while keeping the false negative rate at just 4%. The FC+Dem (AUC = 0.78) and BM+Dem (AUC = 0.81) models also show significant improvements over the baseline. **Figure 6C** provides a visual representation of these results, showing score histograms for the FC+Dem, BM+Dem, and CK+Dem models. For the FC+Dem model, the low-risk threshold achieves 86% accuracy on the control set, a marked improvement compared to the best univariate predictor from the FC set, the RTE-based model, which accurately classifies only 30% of control patients using the same criterion (**Figure 5C**). The relative importance of each feature in the FC+Dem score is depicted in **Figure 6D**, which shows the normalized coefficients of the model. Larger positive (or negative) values indicate stronger positive (or negative) associations with the outcome. All features contribute to the model, as none of the coefficients are zero. The most important negatively associated features are HLA^+^ IF_Mono_, and CD3, while positive associations are observed with age and granulocytes. Interestingly, ranking features by coefficient magnitude does not correspond directly to their univariate AUC rankings (**Figure 5A**). For example, monocyte activation (HLA^+^ IF_Mono_), while the 4^th^-best univariate predictor, is the most important variable in the FC set within the multivariate model. This indicates that the FC+Dem model captures non-trivial interactions between features. From this picture, it is clear that the immune cell state at hospitalization is highly informative of disease progression. However, the most significant feature in the FC+Dem model is age, a proxy variable that does not directly reflect the immune response. This is not the case for the BM+Dem and CK+Dem models (**Figures 6E, F**), validating the observation that cytokines and inflammatory biomarkers hold the most relevant information about disease progression. Finally, we observe that sex and Δt_ons_ show negative associations with outcomes across all models (**Figures 6D–F**), suggesting a higher likelihood of positive outcomes for females, as reported in other studies (42, 43), and for patients hospitalized later after symptom onset.

#### 3.2.1 Outcome prediction across population strata

We conclude our analysis by evaluating the prediction results after stratifying the population by age (≶ 70 years) and Δt_ons_ (≶ 10 days). First, we note that predicting outcomes becomes more challenging for patients over 70 years of age, likely due to increased fragility in this group. Indeed, models using flow cytometry features (FC, FC+Dem) and biomarkers (BM, BM+Dem) have significantly higher AUC for the younger cohort (**Figure 6G**). In contrast, the multivariate predictors using cytokines (CK, CK+Dem) show smaller difference in AUC between the two age groups, reaching AUC = 0.87 for the younger cohort. When stratifying patients by Δt_ons_, predicting outcomes is easier for patients with Δt_ons_ > 10, likely because their more advanced immune response provides clearer prognostic signals. In particular, cytokines-based models exhibit the largest variations in AUC across these strata, with a remarkable AUC = 0.88 for patients with Δt_ons_ > 10 (**Figure 6H**). Therefore, for patients hospitalized later after symptom onset, cytokine levels are highly informative for predicting outcome severity. As expected, adding demographic features (Dem set) offers only minor improvements when stratifying by age and Δt_ons_.

## 4 Discussion

This paper provides a comprehensive description of the immuno-inflammatory response observed in COVID-19 patients before vaccination, thus serving as a case study for a first-time infection by a new virus. Our analysis integrates white blood cell subpopulations, cytokine expression levels, and serological biomarkers measured at hospital admission, offering a multifaceted description of several components of the immune system and inflammatory pathways. Analyzing these features together constitutes one of the core strengths of our contribution, as they are typically studied separately in the literature. From an overall standpoint, we observed that lymphopenia, compromised monocyte function, granulocytosis, and heightened cytokine levels correlated with disease severity and a negative outcome. Older patients exhibited a more compromised immune response characterized by reductions in monocytes, T cells, and B cells, suggestive of immunosenescence. Remarkably, they also showed increased percentages of HLA-DR-positive T cells. Similar patterns were observed in the cohort of asymptomatic outpatients, which, however, maintained higher lymphocyte levels, with only recent thymic emigrants and B cells decreasing with age. Additionally, cytokine expression levels showed a weak positive correlation with age, while mid-regional pro-adrenomedullin (proADM) levels strongly correlated with age, possibly due to underlying chronic low-grade inflammation and endothelial dysfunction that develops with aging. To investigate the temporal behavior of the immune response, we considered the number of days elapsed between symptom onset and hospitalization (Δt_ons_). Our cross-sectional analysis revealed a decrease in the effectiveness of monocytes over time, characterized by an increase in their numbers but a concurrent decrease in their activation level, as measured by the mean intensity fluorescence of HLA-DR. We also observed a reduction in natural killer cells and cytokine levels, higher concentrations of lactate dehydrogenase (LDH), a marker of cell injury, and increased activation of the humoral response, as indicated by B CD19 cells. This pattern mirrors a typical dynamic evolution of the host’s immune response, suggesting that these results should be interpreted longitudinally. On the other hand, patients with different Δt_ons_ may manifest intrinsically different diseases, where some exhibit a rapid and intense response and others undergo a slower but more subtle progression, leading to the need for hospital care at a later stage.

The serum levels of cytokines, whether proinflammatory (IL8, IP10) or immunomodulatory (IL10), emerged as the most crucial aspect of the immuno-inflammatory response for predicting patients’ outcomes. Indeed, cytokine-based logistic regression models substantially outperformed the best baseline index, the 4C score, developed to predict in-hospital mortality for COVID-19 patients. Additionally, pro-adrenomedullin proved to be the best individual predictor for identifying low-risk patients, with a cutoff of ≤ 0.84 nmol/L corresponding to a specificity of 0.44 and a negative predictive value (NPV) of 0.97. Importantly, this cutoff aligns well with those found in other settings (11, 44), underscoring the robustness of this result. However, we shall remark that proADM is weakly correlated with the severity level on the WHO scale, and it may reflect a broader physiological response, not necessarily specific to the severity of COVID-19. Therefore, using proADM alone for risk stratification might not fully address treatment needs specific to COVID-19, such as oxygen-based therapies. In contrast, cytokine expression levels may provide a more targeted tool for identifying low-risk patients, as they show significant correlations with both COVID-19 morbidity and mortality. Notably, when combining cytokine levels with demographic information into a single model, we achieved a remarkable AUC of 0.87. This model also proved very effective in detecting low-risk patients, reliably identifying approximately 60% of individuals who may not require hospitalization, while maintaining a false omission rate of just 4%. Moreover, our analysis revealed that cytokine-based models can be further improved when focusing on patients hospitalized later after symptom onset (Δt_ons_ > 10). This finding, if interpreted longitudinally, suggests that monitoring the evolution of the cytokine levels through follow-up tests could offer valuable predictive insights. Lastly, our analysis shows that flow cytometry features exhibit less predictive power than cytokines (IL6, IP10, IL10, IL8) and biomarkers (LDH, proADM). Nevertheless, features such as recent thymic emigrants and T CD3 cells match the performance of the best baseline predictor, the 4C score. When integrated into a single model, the flow cytometry features outperform the 4C score, leveraging non-trivial relationships between white blood cell subpopulations and thus underscoring the complexity of the immune response. Notably, in the combined model, the mean intensity fluorescence (IF) of monocytes HLA-DR emerges as the most important flow cytometry feature, highlighting the critical role of cell activation in the immune response. Exploring HLA-DR IF in lymphocyte subpopulations could further improve our understanding of the immune response to COVID-19 and other infectious diseases.

### 4.1 Limitations

Despite the valuable insights and contributions provided by this study, several limitations need to be acknowledged. Firstly, our dataset included measurements of cytokine expression levels and serological biomarkers only for subsets of hospitalized patients, with no corresponding measurements for outpatients or healthy individuals. Therefore, our control set only encompassed demographic information and flow cytometry measurements, and we had to rely on reference values for cytokine expressions and inflammatory biomarkers. Moreover, due to the limited availability of information regarding cytokines and biomarkers, we opted to employ multivariate logistic regression models where these features are not combined. This approach aimed to prevent further reduction in dataset size and maintain statistical power in our analyses. Combining all covariates into a single multivariate predictor could potentially improve predictive power, though it would require more advanced feature selection methods to enhance model interpretability (45). Another limitation is the absence of external validation for our predictive models. While our study utilized a large dataset from our hospital, the generalizability of our findings to other healthcare settings, populations, and other infectious diseases remains uncertain. As already reported in the text, it is important to acknowledge the limitation of the Δt_ons_ variable, which relies on the ability of patients to accurately recall when symptom onset occurred. This variable is thus subject to significant variability stemming from differences in symptom perception and memory capabilities of the patients. Furthermore, we remark that our study was conducted within a specific context and timeframe, involving patients admitted to our hospital during the COVID-19 pandemic before the vaccination campaign started. As the understanding of COVID-19 and its management continues to evolve, future studies incorporating diverse populations and settings are needed to validate and expand upon our findings. Finally, like any observational study, our research is subject to potential confounders and unmeasured variables. Despite our best efforts to account and adjust for known factors, there may still be uncontrolled variables that could influence the results.

## 5 Materials and methods

### 5.1 Data collection

This study involves data from patients admitted to the Infectious Disease ward of the Azienda Sanitaria Universitaria Friuli Centrale Santa Maria della Misericordia of Udine, a 1000-bed tertiary-care teaching hospital identified as a regional referral center for COVID-19 patients. The analyzed records were collected from March 2020 to April 2021, covering the first, second, and part of the third pandemic waves. During this period, clinical data from patients admitted for SARS-CoV-2 were included in a retrospective registry, namely the “MAnagement coroNavirus Disease In hospital (MANDI) registry” (authorization of DG, decree n. 957, 10/09/2021). Patients were enrolled in accordance with the Helsinki Declaration. Ethical approval was granted from governance bodies of Friuli Venezia Giulia. The registry included patients admitted to either the infectious disease clinic or the intensive care unit, diagnosed with SARS-CoV2 infection through at least one positive nasopharyngeal swab, confirmed by reverse transcriptase PCR assays. SARS-CoV2 infection detection on nasopharyngeal swabs was based on the presence of unique sequences of virus RNA by nucleic acid amplification in real-time PCR (RT-PCR). The genes investigated were the E gene for screening and the RdRp and N genes for confirmation. RT-PCR was performed using a LightMixr Modular SARS and Wuhan CoV E-gene kit on a LightCyclerr 480 II instrument (Roche, Basel, Switzerland). The specimens were considered positive if the cycle threshold (Ct) value for at least one of the three genes was ≤ 36. The eligible participants were aged 18 years or older and had provided informed consent for the utilization of anonymous clinical data. Hospital admission involved routine inquiries regarding consent for anonymized aggregate data for research purposes, facilitated by the General Electronic Consents (GECO system). Relevant patients’ data were extracted by a team of physicians from the hospital electronic health record (INSIEL, Trieste, Italy), anonymized, and recorded on a cloud-based clinical data management platform (Castor, Netherlands and USA). All patients had not yet been vaccinated against SARS-CoV-2.

### 5.2 Immuno-inflammatory features

Approximately 900 patients underwent lymphocyte and monocyte immunophenotyping within the first 72 hours of hospital admission. For identifying the main lymphocyte and monocyte subpopulations, a multicolor flow cytometry analysis was performed using the following antibodies: CD45 PerCP-Cy5.5, CD3, CD4, CD8, CD19, CD56, CD16, and HLA-DR FITC (BD Biosciences, San Diego, CA, USA). To identify recent thymic emigrant lymphocytes (RTE), the following antibodies were used: CD45 V500, CD3 V450, CD31 PE, CD4 APC (BD Biosciences), and CD45RA PE-Vio770 (Miltenyi Biotec, Germany). Cell acquisition was performed using the automated FACSCanto II instrument (BD Biosciences, San Jose, USA), equipped with 488 nm, 633 nm, and 405 nm lasers. For each tube, 50,000 events were acquired. BD FACS™ 7-color setup beads (BD) were used daily to adjust detector voltages, set fluorescence compensation, and monitor instrument performance. Data analysis was conducted using FACSDiva software (BD Biosciences, San Jose, USA), with cell populations represented through dot-plot graphs.

Lymphocytes were separated using a combination of two gating strategies: a physical gate (FSC-A vs. SSC-A) and a lymphocyte gate (SSC vs. CD45), where the CD45^high^FSC^low^SSC^low^ cell population was selected. T lymphocytes were identified as T CD3^+^ cells (CD3), B lymphocytes as CD3^−^CD19^+^ cells (CD19), and natural killer cells as CD3^−^/CD16^+^CD56^+^ cells (NK). T lymphocytes were further subdivided into helper T lymphocytes, CD3^+^CD4^+^ (CD4), cytotoxic T lymphocytes, CD3^+^CD8^+^ (CD8), activated helper T lymphocytes, CD3^+^CD4^+^HLA-DR^+^, and cytotoxic T lymphocytes, CD3^+^CD8^+^HLA-DR^+^. The latter two were expressed as a percentage of helper T lymphocytes $\left(\text{H}\text{L}{\text{A}}_{\%\text{C}\text{D}4}^{+}\right)$ and cytotoxic T lymphocytes $\left(\text{H}\text{L}{\text{A}}_{\%\text{C}\text{D}8}^{+}\right)$ , respectively. Monocytes were identified based on their physical properties and CD45 expression. The activation of monocyte populations was quantified in terms of the percentage of HLA-DR–positive monocytes $\left(\text{H}\text{L}{\text{A}}_{\%\text{M}\text{o}\text{n}\text{o}}^{+}\right)$ , and by the HLA-DR mean fluorescence intensity, measured in arbitrary units $\left(\text{H}\text{L}{\text{A}}^{+}\text{I}{\text{F}}_{\text{M}\text{o}\text{n}\text{o}}\right)$ . RTEs were defined as the subpopulation of CD3^+^CD4^+^ lymphocytes characterized by coexpression of CD45RA and CD31. The absolute number of lymphocyte and monocyte subpopulations was calculated by an indirect method based on the number of lymphocytes and monocytes determined by the hemocytometer. We refer to the flow cytometry features as the FC set. The same measurements were taken for approximately 370 asymptomatic outpatients and 90 healthy individuals. These two cohorts exhibit matching statistics (**Supplementary Figure 2**), and we employed the outpatients’ data as a control dataset for our analysis. The flow cytometry features provide a comprehensive description of the immune response, encompassing the innate immune system (Mono, NK), the cell-mediated adaptive system (CD4, CD8), and the antibody-mediated adaptive system (CD19). We remark that many of these features are hierarchically related: WBCs consist of lymphocytes, monocytes, and granulocytes. Lymphocytes include CD3, CD19, and NK cells. CD3 cells can be categorized into CD4 and CD8 cells, and RTE cells are a subpopulation of CD4 cells. It is important to consider these relationships to avoid collinearity effects.

In our analysis, we also included a broad panel of systemic inflammatory biomarkers. Among these, we collected data on seven cytokines: interleukin 10 (IL10), IL1-*β* (IL1B), soluble IL2R-*α*/sCD25 (IL2R), interleukin 6 (IL6), interleukin 8 (IL8), chemokine IP10/CXCL10 (IP10), and interferon-*γ* (INF-*γ*). This group of features, referred to as the CK set, includes both pro-inflammatory (e.g., IL6, IL1B) and immunomodulatory molecules (e.g., IL10, IL2R) (14). Cytokines were analyzed using a microfluidic, ultrasensitive, fully automated ELISA method, employing multiplex customized cartridges on the Ella Instrument (R&D Systems, Bio-Techne, USA). Two types of cartridges were used, each capable of measuring 4 cytokines across 16 samples. This technique proved to be highly precise, reproducible (with excellent lot-to-lot consistency), and extremely sensitive, offering up to 4 logs of dynamic range. Detailed information about Limit of Detection and Limit of Quantification of each cytokine can be found on the manufacturer’s website (https://www.biotechne.com). We also collected data on three standardized biomarkers, which we refer to as the BM set: mid-regional pro-adrenomedullin (proADM), lactate dehydrogenase (LDH), and C-reactive protein (CRP). CRP and LDH were tested in serum by a well-established commercial diagnostic method (Elecsys, Roche Diagnostics, Basel, Switzerland), while proADM plasma concentrations were measured in the automated Kryptor analyzer, using the TRACE technology (Kryptor, BRAHMS, Hamburg, Germany).

In addition to the immuno-inflammatory features described above, we collected information on several comorbidities for all patients enrolled in the study, as well as age, sex, and number of days elapsed between symptom onset and hospitalization. We refer to **Supplementary Table 2** for a detailed description of the dataset.

### 5.3 Preprocessing and statistical analysis

Manually filled databases often contain typos, leading to the presence of outliers or attributes with no physical meaning. To minimize the impact of these mistakes and ensure the data’s homogeneity and cleanliness, we implemented the following pre-processing steps: we considered patients with age in the range of 30 to 100 years and Δt_ons_ between 0 and 30 days. We further filtered records to include only patients with a CCI score of less than 7, to mitigate potential confounding factors for the outcome of the patients and ensure a more accurate description of the immune response. High CCI scores indicate the presence of significant comorbidities, which could interfere with the interpretation of immunological data and may lead to an inaccurate representation of the true effects of COVID-19 on the immune system. Finally, we performed a careful inspection of the distributions of features and a conservative outlier removal step. To detect outliers, we employed a power transform from scikit-learn (46) to normalize the data distribution. We then set to NAN data with a z-score of absolute value larger than 3.

Our characterization of the immuno-inflammatory profiles entailed multi-dimensional comparisons among various patient groups. More precisely, we investigated the relationship between features and developed disease severity by stratifying patients according to the WHO scale (**Figure 1**). This scale is based on the World Health Organization’s guidance (16) and categorizes patients into four levels as follows:

1. mild disease: symptomatic patients without pneumonia;
2. moderate disease: patients with clinical signs of pneumonia with no need for oxygen therapy;
3. severe disease: patients with clinical signs of severe pneumonia in need of oxygen therapy;
4. critical disease: patients with oxygenation impairment, acute respiratory syndrome, and/or sepsis.

To detect relevant patterns of the immune response, we also analyzed the rolling median of all features along two axes: the age and Δt_ons_ of the patients. More precisely, we employed a half-window of 15 years to examine age-related variations (**Figure 2**), comparing the median of two age groups: patients aged 40-70 and 70-100 years. Similarly, we used a half-window of 5 days for the Δt_ons_ (**Figure 3**) and compared patients with Δt_ons_ of 1-10 and 11-20 days. The distributions of different population groups were tested using the Mann-Whitney U test.

### 5.4 Outcome prediction

In Sec. 3, we explored the potential of predicting patient outcomes based on their immune response as measured upon hospital admission. To this end, we employed both univariate and multivariate logistic regression (LR) models to predict the joint event of death and/or orotracheal intubation, referred to as the death+OTI outcome. Combining these two events helps to mitigate an important source of bias: patients’ prioritization for invasive and life-saving interventions. Moreover, considering this outcome offers a second advantage: it improves class imbalance, with a negative event observed in approximately 24% of patients, compared to 17% when considering death only (**Supplementary Table 2**). To address the class imbalance, we used logistic regression models from scikit-learn with *l*_2_ penalty and class weights balanced based on class frequency. We evaluated univariate LR models by removing records with missing data, thus capturing the true signal associated with each variable. We randomly split records into train (70%) and test (30%) sets, keeping the class and sex frequencies unchanged, and repeating the process several times to collect performance statistics. For the multivariate models, we considered different subsets of features, controlling for collinearity via the variance inflation factor and domain-based knowledge. Records with more than 50% of missing data in each feature subset were omitted from the analysis. The remaining missing data were replaced via KNN imputation (*K* = 10, neighbors weighted uniformly), and the least significant PCA components (with less than 5% of explained variance) were removed from the data. We combined k-fold cross-validation and grid-search on each train set to select the regularization strength for the *l*_2_ penalty.

We measured the performance of each logistic regression model in two ways. First, we computed the area under the ROC curve (AUC) to quantify the overall predictive power of the classifier. Second, we evaluated the ability of each classifier to detect the largest fraction of low-risk patients, i.e., patients with a positive outcome, while retaining a low false omission rate. Practically, we set a requirement of a negative predictive value (NPV) of 0.97 on the train set, with NPV values above 0.95 considered acceptable on the test set, and we measured the performance in detecting low-risk patients in terms of specificity.

## Data Availability

Publicly available datasets were analyzed in this study. This data can be found here: https://github.com/RiccardoGM/ImmuneResponseCOVID19.

## Supplementary material

Supplementary Table 1 Datasets used for the multivariate predictions. Demographic and clinical characteristics of the datasets employed in the multivariate predictive analysis (see Sec. 3.2). Records with more than 50% missing data in any feature set were excluded from the analysis. The feature sets include flow cytometry variables (FC set), cytokines (CK set), serological inflammatory biomarkers (BM set), and demographic information (Dem set). At least 50% of the Dem set is available for all records. Thus, combining the Dem set with any of the FC, CK, or BM sets does not reduce the dataset size but affects the percentage of missing data. Stratified datasets exclude records with missing information in the stratification variable.

Supplementary Table 2 Extended demographics and clinical characteristics of hospitalized patients and outpatients. Hospitalized patients are also grouped by age, number of days elapsed between symptom onset and hospitalization (Δt_ons_), and WHO level. Dichotomous variables (sex, comorbidities, outcomes) are presented as proportions of occurrences relative to the total number of records with available data. Numerical variables (age, CCI, immune cell counts, cytokines, biomarkers) are expressed in terms of median (Q2), first (Q1) and third (Q3) quartiles, following the format Q2(Q1-Q3). Dashes indicate unavailable data. Demographics: number of records, age, and proportion of male patients. Comorbidities: proportion of patients with obesity, hypertension, cardiovascular disorders (CVDs), dyslipidemia, diabetes, chronic kidney injuries (CKI), solid and hematologic neoplasms (tumor, oncohematology), chronic obstructive pulmonary disease (COPD), autoimmunity, hepatopathy, immunosuppression (primary or secondary). Comorbidities are also summarized in the Charlson Comorbidity Index (CCI). Immune cells measured via flow cytometry: white blood cells (WBC), monocytes (Mono), mean intensity fluorescence of HLA-DR-positive monocytes (HLA^+^IF Mono), percentage of HLA-DR-positive monocytes (HLA^+^ % Mono), lymphocytes (Lymph), T CD3^+^ cells (CD3), percentage of CD3^+^ lymphocytes (CD3% Lymph), HLA-DR-positive T CD3^+^ cells (CD3 HLA^+^), Th CD3^+^CD4^+^ cells (CD4), percentage of CD3^+^CD4^+^ lymphocytes (CD4% Lymph), percentage of HLA-DR-positive CD4 cells (HLA^+^ % CD4), recent thymic emigrants (RTE) and associated percentage of CD4 cells (RTE % CD4), Tc CD3^+^CD8^+^ cells (CD8), percentage of CD3^+^CD8^+^ lymphocytes (CD8% Lymph), percentage of HLA-DR-positive CD8 cells (HLA^+^ % CD8), B CD3^−^CD19^+^ cells (CD19), percentage of CD3^−^CD19^+^ lymphocytes (CD8% Lymph), T NK CD3^−^CD56/CD16^+^ cells (NK), and percentage of CD3^−^CD56/CD16^+^ lymphocytes (NK % Lymph). Cytokines: interferon-*γ* (INF-*γ*), interleukin 10 (IL10), IL1-Beta (IL1B), sIL2R-*α*/sCD25 (IL2R), interleukin 6 (IL6), interleukin 8 (IL8), and interleukin IP10/CXCL10 (IP10). Biomarkers: pro-adrenomedullin (proADM), lactate dehydrogenase (LDH), and C-reactive protein (CRP). Outcomes: proportion of patients who developed infectious complications, underwent orotracheal intubation (OTI), died (death), or experienced either OTI or death (death+OTI).

Supplementary Figure 1 Flow cytometry features of outpatients and inpatients vs age. **(A)** Running median of flow cytometry variables with a 15-year half-window. Shaded areas represent the first to third running quartiles. Significant changes in distribution between ages 35 and 65 are expressed as the percentage increase or decrease of the median. **(B)** Distribution of flow cytometry variables stratified by age and cohort (inpatients vs outpatients). Boxes span the first to third quartiles, with the horizontal bar indicating the median. Median comparisons on the right of each plot are quantified as the percentage change between inpatients and outpatients. **(C)** Age histograms of inpatients and outpatients. Measurement units: white blood cells (WBC) and related subpopulations (Lymph, Mono, CD3, CD4, CD8, NK, CD19, RTE) are expressed in U/*µ*L.

Supplementary Figure 2 Flow cytometry feature distributions for inpatients, outpatients, and the healthy cohort. To ensure comparability, data for all variables were scaled and shifted to standardized statistics within the healthy population. Statistical comparisons between the healthy cohort and outpatients/inpatients were conducted using the Mann–Whitney U test. Proportions of male patients were compared using the z-test. The statistical significance of each comparison is provided by the p-value.

## References

[1] Kowsar R Rahimi AM Sroka M Mansouri A Sadeghi K Bonakdar E. Risk of mortality in COVID-19 patients: a meta- and network analysis. Sci Rep. (2023) 13:2138. doi: 10.1038/s41598-023-29364-8 36747045 PMC9901837

[2] C.-M.-o. B. A. (Consortium. A blood atlas of COVID-19 defines hallmarks of disease severity and specificity. Cell. (2022) 185:916–93858. doi: 10.1016/j.cell.2022.01.012. issn: 1097-4172. 35216673 PMC8776501

[3] Vijayakumar B Boustani K Ogger PP Papadaki A Tonkin J Orton CM. Immuno-proteomic profiling reveals aberrant immune cell regulation in the airways of individuals with ongoing post-COVID-19 respiratory disease. Immunity. (2022) 55:542–5565. doi: 10.1016/j.immuni.2022.01.017. issn: 1097-4180. 35151371 PMC8789571

[4] Rello J Valenzuela-Sánchez F Ruiz-Rodriguez M Moyano S. Sepsis: A review of advances in management. Adv Ther. (2017) 34:2393–411. doi: 10.1007/s12325-017-0622-8 PMC5702377 29022217

[5] Herminghaus A Osuchowski MF. How sepsis parallels and differs from COVID-19. eBioMedicine. (2022) 86:104355. doi: 10.1016/j.ebiom.2022.104355. issn: 2352-3964. 36470836 PMC9718536

[6] Karakike E Giamarellos-Bourboulis EJ Kyprianou M Fleischmann-Struzek C Pletz MW Netea MG. Coronavirus disease 2019 as cause of viral sepsis: A systematic review and meta-analysis. Crit Care Med. (2021) 49:2042–57. doi: 10.1097/CCM.0000000000005195. issn: 1530-0293. PMC8594513 34259663

[7] Hamers L Kox M Pickkers P. Sepsis-induced immunoparalysis: mechanisms, markers, and treatment options. Minerva Anestesiol. (2015) 81:426–39. issn: 1827-1596. 24878876

[8] Bruscoli S Puzzovio PG Zaimi M Tiligada K Levi-Schaffer F Riccardi C. Glucocorticoids and COVID-19. Pharmacol Res. (2022) 185:106511. doi: 10.1016/j.phrs.2022.106511 36243331 PMC9556882

[9] British Medical Journal Publishing Group. Risk stratification of patients admitted to hospital with covid-19 using the ISARIC WHO Clinical Characterisation Protocol: development and validation of the 4C Mortality Score. BMJ. (2020) 371:m4334. doi: 10.1136/bmj.m4334. issn: 1756-1833. 33187971 PMC7662116

[10] Crocker-Buque T Myles J Brentnall A Gabe R Duffy S Williams S. Using ISARIC 4C mortality score to predict dynamic changes in mortality risk in COVID-19 patients during hospital admission. PloS One. (2022) 17:e0274158. doi: 10.1371/journal.pone.0274158. issn: 1932-6203. 36223373 PMC9555674

[11] Sozio E Moore NA Fabris M Ripoli A Rumbolo F Minieri M. Identification of COVID-19 patients at risk of hospital admission and mortality: a European multicentre retrospective analysis of mid-regional pro-adrenomedullin. Respir Res. (2022) 23:221. doi: 10.1186/s12931-022-02151-1. issn: 1465-993X. 36031619 PMC9420187

[12] Saeed K Legramante JM Angeletti S Curcio F Miguens I Poole S. Mid-regional pro-adrenomedullin as a supplementary tool to clinical parameters in cases of suspicion of infection in the emergency department. Expert Rev Mol Diagn. (2021) 21:397–404. doi: 10.1080/14737159.2021.1902312. issn: 1744-8352. 33736553

[13] Deana C Vetrugno L Fabris M Curcio F Sozio E Tascini C. Pericardial cytokine “Storm” in a COVID-19 patient: the confirmation of a hypothesis. Inflammation. (2022) 45:1–5. doi: 10.1007/s10753-021-01563-3. issn: 1573-2576. 34533672 PMC8446479

[14] Fabris M Del Ben F Sozio E Beltrami AP Cifù A Bertolino G. Cytokines from bench to bedside: A retrospective study identifies a definite panel of biomarkers to early assess the risk of negative outcome in COVID-19 patients. Int J Mol Sci. (2022) 23:4830. doi: 10.3390/ijms23094830. issn: 1422-0067. 35563218 PMC9101406

[15] Beltrami AP De Martino M Dalla E Malfatti MC Caponnetto F Codrich M. Combining deep phenotyping of serum proteomics and clinical data via machine learning for COVID-19 biomarker discovery. Int J Mol Sci. (2022) 23:9161. doi: 10.3390/ijms23169161. issn: 1422-0067. 36012423 PMC9409308

[16] W. H. (hq). Clinical management of COVID-19. In: World Health Organization (2020). p. 1–62. Available at: https://www.who.int/publications/i/item/clinical-management-of-covid-19/.

[17] Charlson ME Pompei P Ales KL MacKenzie CR. A new method of classifying prognostic comorbidity in longitudinal studies: Development and validation. J Chronic Dis. (1987) 40:373–83. doi: 10.1016/0021-9681(87)90171-8. issn: 0021-9681. 3558716

[18] Mervyn Singer M. The third international consensus definitions for sepsis and septic shock (Sepsis-3). JAMA. (2016) 315:801–10. doi: 10.1001/jama.2016.0287. issn: 0098-7484. PMC4968574 26903338

[19] Ferreira FL Bota DP Bross A Mélot C Vincent JL. Serial evaluation of the SOFA score to predict outcome in critically ill patients. JAMA. (2001) 286:1754–8. doi: 10.1001/jama.286.14.1754. issn: 0098-7484. 11594901

[20] Kostakis I Smith GB Prytherch D Meredith P Price C Chauhan A. The performance of the National Early Warning Score and National Early Warning Score 2 in hospitalised patients infected by the severe acute respiratory syndrome coronavirus 2 (SARS-CoV-2). Resuscitation. (2021) 159:150–7. doi: 10.1016/j.resuscitation.2020.10.039. issn: 1873-1570. PMC7648887 33176170

[21] Smith GB Redfern OC Pimentel MA Gerry S Collins GS Malycha J. The national early warning score 2 (NEWS2). Clin Med. (2019) 19:260. doi: 10.7861/clinmedicine.19-3-260 PMC6542226 31092526

[22] Haimovich AD Ravindra NG Stoytchev S Young HP Wilson FP van Dijk D. Development and validation of the quick COVID-19 severity index: A prognostic tool for early clinical decompensation. Ann Emerg Med. (2020) 76:442–53. doi: 10.1016/j.annemergmed.2020.07.022. issn: 0196-0644. PMC7373004 33012378

[23] Gupta RK Harrison EM Ho A Docherty AB Knight SR van Smeden M. Development and validation of the ISARIC 4C Deterioration model for adults hospitalised with COVID-19: a prospective cohort study. Lancet Respir Med. (2021) 9:349–59. doi: 10.1016/S2213-2600(20)30559-2. issn: 2213-2600. PMC7832571 33444539

[24] Knight SR Ho A Pius R Buchan I Carson G Drake TM. Risk stratification of patients admitted to hospital with covid-19 using the ISARIC WHO Clinical Characterisation Protocol: development and validation of the 4C Mortality Score. BMJ. (2020) 370:m3339. doi: 10.1136/bmj.m3339. issn: 1756-1833. 32907855 PMC7116472

[25] Jones A Pitre T Junek M Kapralik J Patel R Feng E. External validation of the 4C mortality score among COVID-19 patients admitted to hospital in Ontario, Canada: a retrospective study. Sci Rep. (2021) 11:1–7. doi: 10.1038/s41598-021-97332-1. issn: 2045-2322. 34545103 PMC8452633

[26] Li J Zhang K Zhang Y Gu Z Huang C. Neutrophils in COVID-19: recent insights and advances. Virol J. (2023) 20:169. doi: 10.1186/s12985-023-02116-w 37533131 PMC10398943

[27] Del Giudice M Gangestad SW. Rethinking IL-6 and CRP: Why they are more than inflammatory biomarkers, and why it matters. Brain Behav Immun. (2018) 70:61–75. doi: 10.1016/j.bbi.2018.02.013. issn: 0889-1591. 29499302

[28] Gauldie J Richards C Harnish D Lansdorp P Baumann H. Interferon beta 2/B-cell stimulatory factor type 2 shares identity with monocyte-derived hepatocyte-stimulating factor and regulates the major acute phase protein response in liver cells. Proc Natl Acad Sci USA. (1987) 84:7251–5. doi: 10.1073/pnas.84.20.7251. issn: 0027-8424. PMC299269 2444978

[29] Hadjadj J Yatim N Barnabei L Corneau A Boussier J Smith N. Impaired type I interferon activity and inflammatory responses in severe COVID-19 patients. Science. (2020) 369:718–24. doi: 10.1126/science.abc6027. issn: 1095-9203. PMC7402632 32661059

[30] Boroujeni ME Sekrecka A Antonczyk A Hassani S Sekrecki M Nowicka H. Dysregulated interferon response and immune hyperactivation in severe COVID-19: targeting STATs as a novel therapeutic strategy. Front Immunol. (2022) 13:888897. doi: 10.3389/fimmu.2022.888897 35663932 PMC9156796

[31] Chen LYC Quach TTT. COVID-19 cytokine storm syndrome: a threshold concept. Lancet Microbe. (2021) 2:e49–50. doi: 10.1016/S2666-5247(20)30223-8. issn: 2666-5247. PMC7906728 33655230

[32] Leisman DE Ronner L Pinotti R Taylor MD Sinha P Calfee CS. Cytokine elevation in severe and critical COVID-19: a rapid systematic review, meta-analysis, and comparison with other inflammatory syndromes. Lancet Respir Med. (2020) 8:1233–44. doi: 10.1016/S2213-2600(20)30404-5. issn: 2213-2619. PMC7567529 33075298

[33] Sozio E Tascini C Fabris M D'Aurizio F De Carlo C Graziano E. MR-proADM as prognostic factor of outcome in COVID-19 patients. Sci Rep. (2021) 11:5121. doi: 10.1038/s41598-021-84478-1. issn: 2045-2322. 33664308 PMC7933259

[34] Romero Starke K Reissig D Petereit-Haack G Schmauder S Nienhaus A Seidler A. The isolated effect of age on the risk of COVID-19 severe outcomes: a systematic review with meta-analysis. BMJ Global Health. (2021) 6:e006434. doi: 10.1136/bmjgh-2021-006434 PMC8678541 34916273

[35] Haynes L. Aging of the immune system: research challenges to enhance the health span of older adults”. In. Front Aging. (2020) 1:602108. doi: 10.3389/fragi.2020.602108. issn: 2673-6217. 35822168 PMC9261332

[36] Saavedra D Añé-Kourí AL Barzilai N Caruso C Cho KH Fontana L. Aging and chronic inflammation: highlights from a multidisciplinary workshop. Immun Ageing. (2023) 20:1–10. doi: 10.1186/s12979-023-00352-w. issn: 1742-4933. 37291596 PMC10248980

[37] Brouwers FP de Boer RA van der Harst P Struck J de Jong PE de Zeeuw D. Influence of age on the prognostic value of mid-regional pro-adrenomedullin in the general population. Heart. (2012) 98:1348–53. doi: 10.1136/heartjnl-2012-302390. issn: 1355-6037. 22821276

[38] Brennan K Zheng J. Interleukin 8. In: xPharm: the Comprehensive Pharmacology Reference. Elsevier, Walthm, MA, USA (2007), ISBN: isbn: 978-0-08-055232-3. p. 1–4. doi: 10.1016/B978-008055232-3.61916-6

[39] Stanic B van de Veen W Wirz OF Rückert B Morita H Söllner S. IL-10-overexpressing B cells regulate innate and adaptive immune responses. J Allergy Clin Immunol. (2015) 135:771–808. doi: 10.1016/j.jaci.2014.07.041. issn: 1097-6825. 25240783

[40] Wu Z McGoogan JM. Characteristics of and important lessons from the coronavirus disease 2019 (COVID-19) outbreak in China: summary of a report of 72 314 cases from the chinese center for disease control and prevention. JAMA. (2020) 323:1239–42. doi: 10.1001/jama.2020.2648. issn: 1538-3598. 32091533

[41] Onder G Rezza G Brusaferro S. Case-fatality rate and characteristics of patients dying in relation to COVID-19 in Italy. JAMA. (2020) 323:1775–6. doi: 10.1001/jama.2020.4683. issn: 1538-3598. 32203977

[42] Peckham H de Gruijter NM Raine C Radziszewska A Ciurtin C Wedderburn LR. Male sex identified by global COVID-19 meta-analysis as a risk factor for death and ITU admission. Nat Commun. (2020) 11:6317. doi: 10.1038/s41467-020-19741-6. issn: 2041-1723. 33298944 PMC7726563

[43] Kragholm K Andersen MP Gerds TA Butt JH Østergaard L Polcwiartek C. Association between male sex and outcomes of Coronavirus Disease 2019 (Covid-19) – a Danish nationwide, register-based study. Clin Infect Dis. (2024) 16:e70789. doi: 10.1093/cid/ciaa924 PMC7454435 32634827

[44] Saeed K Wilson DC Bloos F Schuetz P van der Does Y Melander O. The early identification of disease progression in patients with suspected infection presenting to the emergency department: a multi-centre derivation and validation study. Crit Care. (2019) 23:40. doi: 10.1186/s13054-019-2329-5. issn: 1466-609X. 30736862 PMC6368690

[45] Wild R Sozio E Margiotta RG Dellai F Acquasanta A Del Ben F. Maximally informative feature selection using Information Imbalance: Application to COVID-19 severity prediction. Sci Rep. (2024) 14:1–11. doi: 10.1038/s41598-024-61334-6. issn: 2045-2322. 38730063 PMC11087653

[46] Pedregosa F Varoquaux Gramfort A Michel V Thirion B Grisel O. Scikit-learn: machine learning in python. J Mach Learn Res. (2011) 12:2825–30.

