# Supplementary material for: Investigating the relationship between the immune response and the severity of COVID-19: a large-cohort retrospective study
Source: Front Immunol. 2025 Jan 8;15:1452638. doi: 10.3389/fimmu.2024.1452638 (PMC11750771; doi:10.3389/fimmu.2024.1452638)
Supplement: Supplementary Table 1 — Datasets used for the multivariate predictions. Demographic and clinical characteristics of the datasets employed in the multivariate predictive analysis (see Sec. 3.2). Records with more than 50% missing data in any feature set were excluded from the analysis. The feature sets include flow cytometry variables (FC set), cytokines (CK set), serological inflammatory biomarkers (BM set), and demographic information (Dem set). At least 50% of the Dem set is available for all records. Thus, combining the Dem set with any of the FC, CK, or BM sets does not reduce the dataset size but affects the percentage of missing data. Stratified datasets exclude records with missing information in the stratification variable. [file SupplementaryFile1.pdf]

# A Supplementary material

Table 1: ***Datasets used for the multivariate predictions***. Demographic and clinical characteristics of the datasets employed in the multivariate predictive analysis (see Sec. 3). Records with more than 50% missing data in any feature set were excluded from the analysis. The feature sets include flow cytometry variables (FC set), cytokines (CK set), serological inflammatory biomarkers (BM set), and demographic information (Dem set). At least 50% of the Dem set is available for all records. Thus, combining the Dem set with any of the FC, CK, or BM sets does not reduce the dataset size but affects the percentage of missing data. Stratified datasets exclude records with missing information in the stratification variable.

**all patients**: $30 \leq$ age $\leq 100$, $0 \leq \Delta t_{ons} \leq 30$

| set | N | sex (female) | age [$Q_2(Q_1$-$Q_3)$] | $\Delta t_{ons}$ [$Q_2(Q_1$-$Q_3)$] | CCI < 2 | NANs (+Dem) | OTI+death |
|---|---|---|---|---|---|---|---|
| FC | 730 | 32.6% | 68 (58-76) | 9 (7-12) | 19.5% | 1.3% (2.1%) | 24.9% |
| CK | 297 | 34.3% | 67 (56-77) | 9 (7-12) | 22.2% | 0.9% (2.1%) | 23.6% |
| BM | 539 | 35.1% | 68 (58-76) | 9 (7-12) | 20.0% | 6.9% (5.3%) | 20.8% |

**younger cohort**: $30 \leq$ age $\leq 70$, $0 \leq \Delta t_{ons} \leq 30$

| set | N | sex (female) | age [$Q_2(Q_1$-$Q_3)$] | $\Delta t_{ons}$ [$Q_2(Q_1$-$Q_3)$] | CCI < 2 | NANs (+Dem) | OTI+death |
|---|---|---|---|---|---|---|---|
| FC | 388 | 26.0% | 59 (53-64) | 9 (7-12) | 36.6% | 1.8% (2.1%) | 16.8% |
| CK | 169 | 26.6% | 57 (52-63) | 9 (7-12) | 39.1% | 0.9% (1.8%) | 16.6% |
| BM | 285 | 29.5% | 59 (52-64) | 9 (7-12) | 37.9% | 6.9% (4.9%) | 14.0% |

**older cohort**: $70 <$ age $\leq 100$, $0 \leq \Delta t_{ons} \leq 30$

| set | N | sex (female) | age [$Q_2(Q_1$-$Q_3)$] | $\Delta t_{ons}$ [$Q_2(Q_1$-$Q_3)$] | CCI < 2 | NANs (+Dem) | OTI+death |
|---|---|---|---|---|---|---|---|
| FC | 342 | 40.1% | 77 (73-81) | 9 (7-12) | 33.6% | 1.0% (2.2%) | 34.2% |
| CK | 128 | 44.5% | 78 (74-84) | 9 (6-12) | 25.8% | 0.6% (2.4%) | 32.8% |
| BM | 254 | 41.3% | 77 (73-82) | 9 (6-12) | 33.5% | 6.8% (5.7%) | 28.3% |

**early arrival**: $30 \leq$ age $\leq 100$, $0 \leq \Delta t_{ons} \leq 10$

| set | N | sex (female) | age [$Q_2(Q_1$-$Q_3)$] | $\Delta t_{ons}$ [$Q_2(Q_1$-$Q_3)$] | CCI < 2 | NANs (+Dem) | OTI+death |
|---|---|---|---|---|---|---|---|
| FC | 405 | 31.9% | 67 (57-76) | 8 (6-9) | 21.5% | 1.6% (1.0%) | 26.9% |
| CK | 168 | 32.1% | 66 (56-77) | 7 (5-9) | 24.4% | 0.6% (0.4%) | 26.8% |
| BM | 308 | 33.8% | 66 (57-76) | 8 (5-9) | 22.4% | 7.6% (3.8%) | 22.1% |

**late arrival**: $30 \leq$ age $\leq 100$, $10 < \Delta t_{ons} \leq 30$

| set | N | sex (female) | age [$Q_2(Q_1$-$Q_3)$] | $\Delta t_{ons}$ [$Q_2(Q_1$-$Q_3)$] | CCI < 2 | NANs (+Dem) | OTI+death |
|---|---|---|---|---|---|---|---|
| FC | 250 | 30.8% | 68 (59-74) | 13 (12-15) | 18.8% | 0.9% (0.6%) | 22.4% |
| CK | 93 | 32.3% | 64 (56-74) | 13 (12-15) | 21.5% | 1.5% (0.9%) | 17.2% |
| BM | 175 | 34.9% | 67 (58-74) | 13 (12-15) | 19.4% | 5.7% (2.9%) | 16.0% |

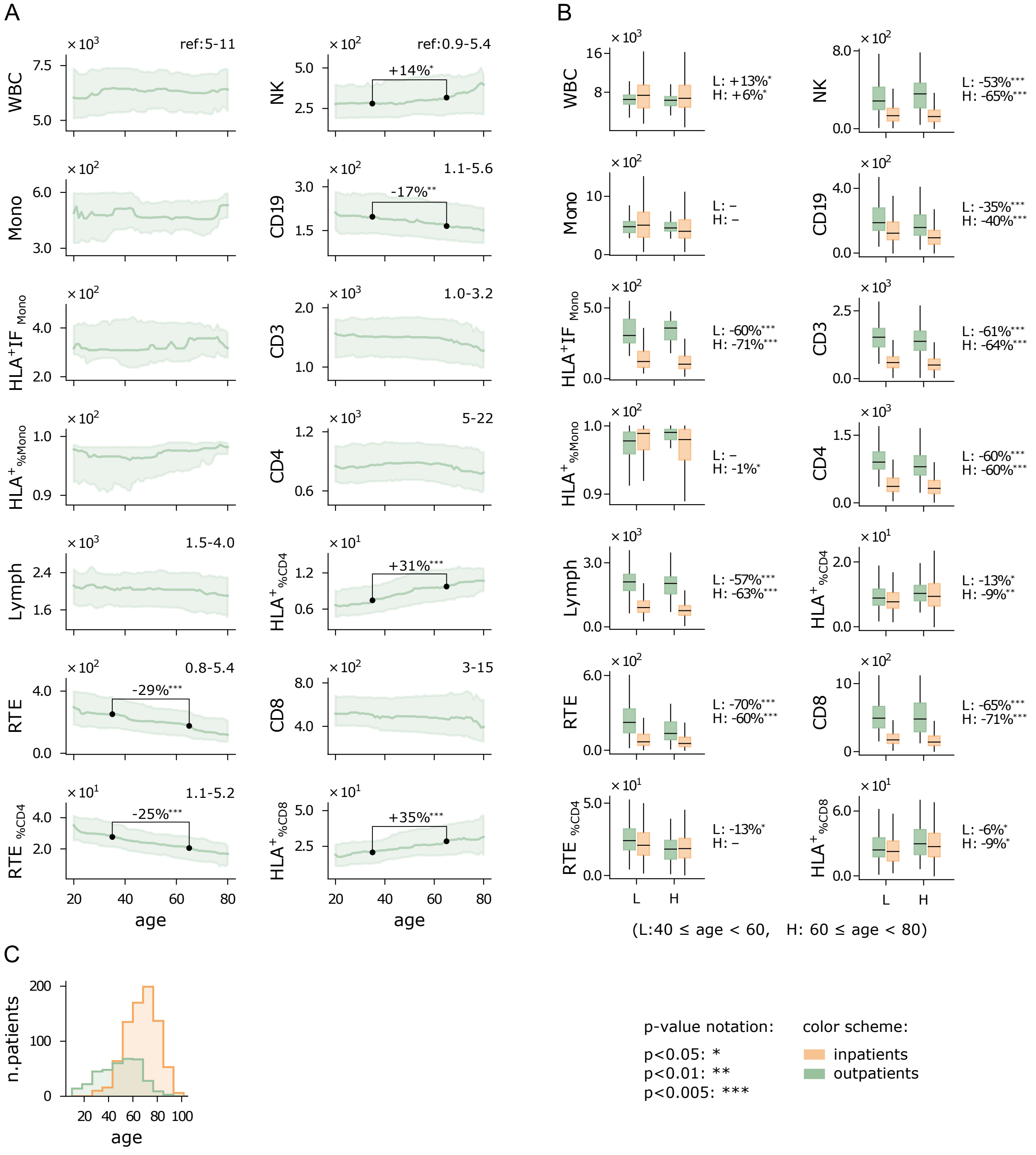


Figure 1: ***Flow cytometry features of outpatients and inpatients vs age.*** (**A**) Running median of flow cytometry variables with a 15-year half-window. Shaded areas represent the first to third running quartiles. Significant changes in distribution between ages 35 and 65 are expressed as the percentage increase or decrease of the median. (**B**) Distribution of flow cytometry variables stratified by age and cohort (inpatients vs outpatients). Boxes span the first to third quartiles, with the horizontal bar indicating the median. Median comparisons on the right of each plot are quantified as the percentage change between inpatients and outpatients. (**C**) Age histograms of inpatients and outpatients. Measurement units: white blood cells (WBC) and related subpopulations (Lymph, Mono, CD3, CD4, CD8, NK, CD19, RTE) are expressed in U/$\mu$L.

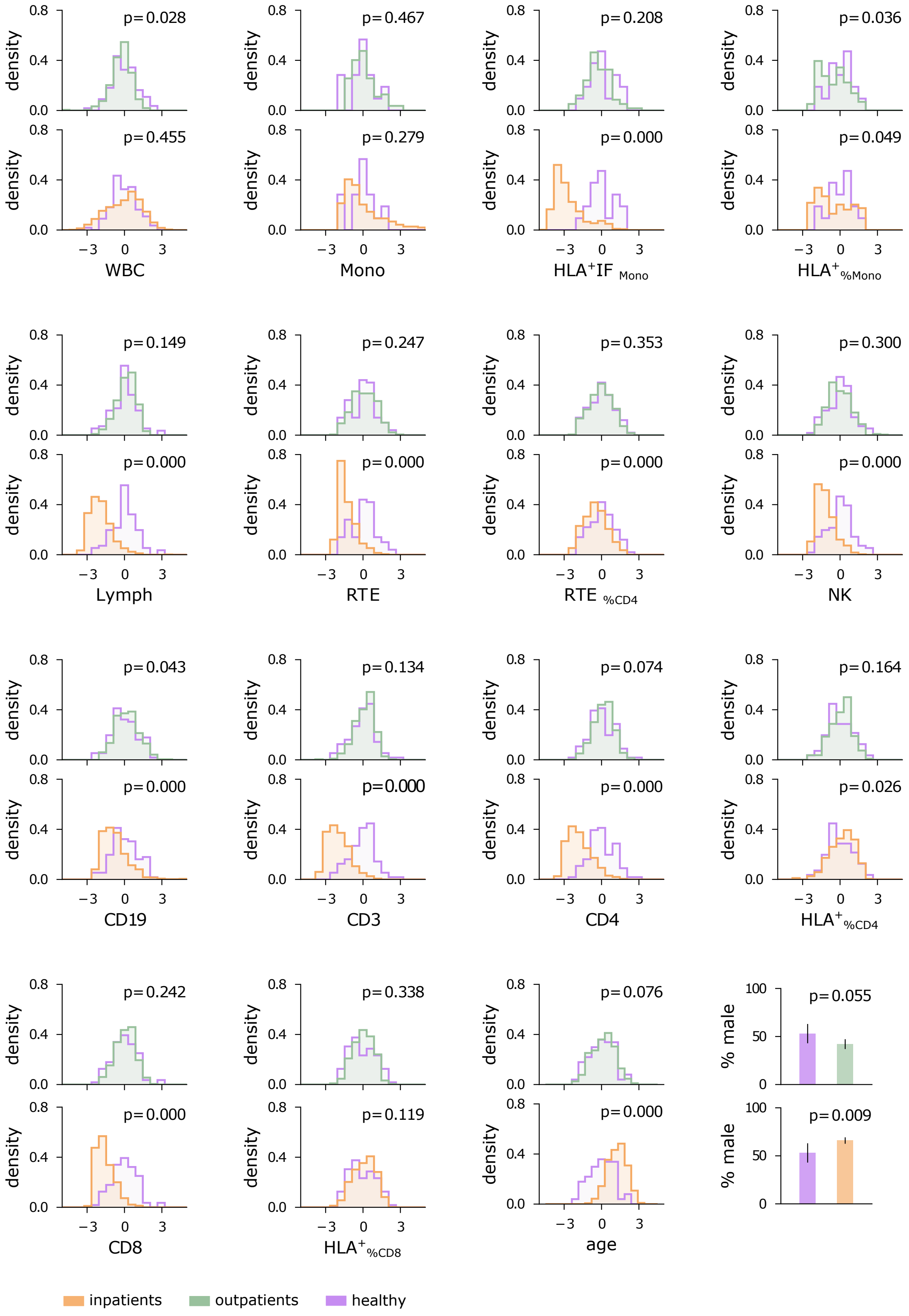


Figure 2: ***Flow cytometry feature distributions for inpatients, outpatients, and the healthy cohort***. To ensure comparability, data for all variables were scaled and shifted to standardized statistics within the healthy population. Statistical comparisons between the healthy cohort and outpatients/inpatients were conducted using the Mann–Whitney U test. Proportions of male patients were compared using the z-test. The statistical significance of each comparison is provided by the p-value.

Table 2: ***Extended demographics and clinical characteristics*** of hospitalized patients and outpatients. Hospitalized patients are also grouped by age, number of days elapsed between symptom onset and hospitalization ($\Delta t_{ons}$), and WHO level. Dichotomous variables (sex, comorbidities, outcomes) are presented as proportions of occurrences relative to the total number of records with available data. Numerical variables (age, CCI, immune cell counts, cytokines, biomarkers) are expressed in terms of median (Q2), first (Q1) and third (Q3) quartiles, following the format Q2(Q1-Q3). Dashes indicate unavailable data.

Demographics: number of records, age, and proportion of male patients.

Comorbidities: proportion of patients with obesity, hypertension, cardiovascular disorders (CVDs), dyslipidemia, diabetes, chronic kidney injuries (CKI), solid and hematologic neoplasms (tumor, oncohematology), chronic obstructive pulmonary disease (COPD), autoimmunity, hepatopathy, immunosuppression (primary or secondary). Comorbidities are also summarized in the Charlson Comorbidity Index (CCI).

Immune cells measured via flow cytometry: white blood cells (WBC), monocytes (Mono), mean intensity fluorescence of HLA-DR-positive monocytes ($HLA^{+}$IF Mono), percentage of HLA-DR-positive monocytes ($HLA^{+}$ % Mono), lymphocytes (Lymph), T $CD3^{+}$ cells (CD3), percentage of $CD3^{+}$ lymphocytes (CD3 % Lymph), HLA-DR-positive T $CD3^{+}$ cells (CD3 $HLA^{+}$), Th $CD3^{+}CD4^{+}$ cells (CD4), percentage of $CD3^{+}CD4^{+}$ lymphocytes (CD4 % Lymph), percentage of HLA-DR-positive CD4 cells ($HLA^{+}$ % CD4), recent thymic emigrants (RTE) and associated percentage of CD4 cells (RTE % CD4), Tc $CD3^{+}CD8^{+}$ cells (CD8), percentage of $CD3^{+}CD8^{+}$ lymphocytes (CD8 % Lymph), percentage of HLA-DR-positive CD8 cells ($HLA^{+}$ % CD8), B $CD3^{-}CD19^{+}$ cells (CD19), percentage of $CD3^{-}CD19^{+}$ lymphocytes (CD8 % Lymph), T NK $CD3^{-}CD56/CD16^{+}$ cells (NK), and percentage of $CD3^{-}CD56/CD16^{+}$ lymphocytes (NK % Lymph). Cytokines: interferon-$\gamma$ (INF-$\gamma$), interleukin 10 (IL10), IL1-Beta (IL1B), sIL2R-$\alpha$/sCD25 (IL2R), interleukin 6 (IL6), interleukin 8 (IL8), and interleukin IP10/CXCL10 (IP10).

Biomarkers: pro-adrenomedullin (proADM), lactate dehydrogenase (LDH), and C-reactive protein (CRP).

Outcomes: proportion of patients who developed infectious complications, underwent orotracheal intubation (OTI), died (death), or experienced either OTI or death (death+OTI).

| demographics | age < 70 | age ≥ 70 | $\Delta t_{ons} \leq 10$ | $\Delta t_{ons} > 10$ | WHO ≤ 2 | WHO > 2 | all inpatients | outpatients |
|---|---|---|---|---|---|---|---|---|
| number | 433 | 357 | 441 | 263 | 263 | 452 | 790 | 367 |
| age | 60 (54-65) | 78 (74-82) | 67 (58-76) | 69 (59-75) | 66 (56-76) | 69 (60-77) | 68 (59-77) | 51 (36-61) |
| sex (male) | 311/433 (72%) | 212/357 (59%) | 295/441 (67%) | 179/263 (68%) | 148/263 (56%) | 318/452 (70%) | 523/790 (66%) | 153/367 (42%) |

| comorbidities | age < 70 | age ≥ 70 | $\Delta t_{ons} \leq 10$ | $\Delta t_{ons} > 10$ | WHO ≤ 2 | WHO > 2 | all inpatients | outpatients |
|---|---|---|---|---|---|---|---|---|
| obesity | 230/364 (63%) | 143/269 (53%) | 196/343 (57%) | 144/228 (63%) | 127/217 (59%) | 226/356 (63%) | 373/633 (59%) | - |
| hypertension | 169/428 (39%) | 234/351 (67%) | 227/433 (52%) | 134/261 (51%) | 119/257 (46%) | 241/448 (54%) | 403/779 (52%) | - |
| CVDs | 90/427 (21%) | 156/352 (44%) | 145/434 (33%) | 65/261 (25%) | 84/261 (32%) | 139/444 (31%) | 246/779 (32%) | - |
| dyslipidemia | 76/400 (19%) | 95/326 (29%) | 92/403 (23%) | 60/245 (24%) | 54/244 (22%) | 102/408 (25%) | 171/726 (24%) | - |
| diabetes | 62/427 (15%) | 71/353 (20%) | 72/437 (16%) | 45/260 (17%) | 40/260 (15%) | 77/446 (17%) | 133/780 (17%) | - |
| CKI | 25/430 (6%) | 28/354 (8%) | 31/437 (7%) | 15/262 (6%) | 20/261 (8%) | 31/449 (7%) | 53/784 (7%) | - |
| tumor | 14/427 (3%) | 18/354 (5%) | 19/434 (4%) | 8/261 (3%) | 13/260 (5%) | 17/447 (4%) | 32/781 (4%) | - |
| oncohematology | 16/429 (4%) | 14/353 (4%) | 16/434 (4%) | 9/262 (3%) | 11/261 (4%) | 18/448 (4%) | 30/782 (4%) | - |
| COPD | 13/425 (3%) | 28/350 (8%) | 28/433 (6%) | 10/257 (4%) | 9/259 (3%) | 28/444 (6%) | 41/775 (5%) | - |
| autoimmunity | 18/427 (4%) | 23/353 (7%) | 25/434 (6%) | 12/260 (5%) | 16/259 (6%) | 21/447 (5%) | 41/780 (5%) | - |
| hepatopathy | 24/430 (6%) | 9/355 (3%) | 21/437 (5%) | 9/262 (3%) | 12/262 (5%) | 17/449 (4%) | 33/785 (4%) | - |
| immunosuppression | 18/428 (4%) | 12/353 (3%) | 19/435 (4%) | 8/261 (3%) | 10/258 (4%) | 16/449 (4%) | 30/781 (4%) | - |
| CCI | 2 (1-3) | 4 (3-5) | 3 (2-4) | 3 (2-4) | 3 (1-4) | 3 (2-4) | 3 (2-4) | - |

| immune cells | age < 70 | age ≥ 70 | $\Delta t_{ons} \leq 10$ | $\Delta t_{ons} > 10$ | WHO ≤ 2 | WHO > 2 | all inpatients | outpatients |
|---|---|---|---|---|---|---|---|---|
| WBC ($10^3$ U/$\mu$L) | 7.0 (4.8-9.6) | 6.4 (4.8-9.5) | 6.2 (4.2-8.6) | 8.0 (5.9-10.4) | 6.1 (4.1-8.5) | 7.7 (5.4-10.4) | 6.8 (4.8-9.5) | 6.4 (5.3-7.3) |
| Mono ($10^2$ U/$\mu$L) | 4.6 (2.9-7.1) | 3.9 (2.8-5.8) | 3.8 (2.5-5.7) | 5.1 (3.3-7.0) | 4.3 (2.9-6.7) | 4.3 (2.9-6.4) | 4.3 (2.9-6.3) | 4.7 (3.9-5.9) |
| HLA$^+$ IF Mono ($10^2$ U) | 1.1 (0.8-1.7) | 1.0 (0.7-1.6) | 1.1 (0.8-1.8) | 0.9 (0.7-1.4) | 1.4 (0.8-2.2) | 0.9 (0.6-1.3) | 1.1 (0.7-1.7) | 3.2 (2.7-4.2) |
| HLA$^+$ % Mono | 98.7 (96.0-99.5) | 98.0 (94.0-99.4) | 98.7 (96.0-99.6) | 98.0 (93.7-99.1) | 99.0 (97.0-99.7) | 98.0 (94.0-99.3) | 98.4 (95.0-99.5) | 98.7 (96.5-99.2) |
| Lymph ($10^2$ U/$\mu$L) | 8.5 (6.1-11.5) | 7.0 (4.8-9.4) | 7.9 (5.4-10.1) | 7.2 (5.0-10.3) | 9.0 (6.5-11.9) | 7.0 (5.0-9.8) | 7.9 (5.5-10.5) | 20.4 (16.5-24.0) |
| CD3 ($10^2$ U/$\mu$L) | 5.6 (3.8-8.1) | 4.6 (2.9-6.8) | 5.1 (3.3-7.1) | 4.9 (3.1-7.3) | 6.0 (4.3-8.6) | 4.6 (2.9-6.7) | 5.1 (3.3-7.4) | 15.0 (11.4-18.2) |
| CD3 % Lymph | 68.0 (59.0-76.0) | 67.0 (57.0-75.0) | 67.0 (57.0-75.0) | 66.0 (58.0-76.0) | 70.0 (62.0-77.0) | 65.0 (56.0-74.0) | 68.0 (58.0-76.0) | 74.0 (67.0-79.8) |
| CD3 HLA$^+$ ($10^1$ U/$\mu$L) | 7.2 (4.2-11.8) | 6.8 (3.8-11.7) | 6.7 (3.7-10.4) | 6.4 (3.9-11.6) | 8.6 (4.9-14.4) | 6.3 (3.6-9.8) | 7.1 (4.0-11.8) | 20.1 (13.4-30.4) |
| CD4 ($10^2$ U/$\mu$L) | 3.6 (2.4-5.4) | 2.9 (1.8-4.4) | 3.2 (2.1-4.7) | 3.1 (2.0-5.1) | 3.9 (2.5-5.9) | 2.9 (1.9-4.5) | 3.3 (2.1-5.1) | 8.6 (6.8-10.7) |
| CD4 % Lymph | 43.0 (35.0-52.0) | 43.0 (33.0-51.0) | 42.0 (33.0-51.0) | 44.0 (35.0-52.0) | 45.0 (36.0-54.0) | 42.0 (33.0-50.2) | 43.0 (34.0-51.8) | 44.0 (39.0-49.0) |
| HLA$^+$ % CD4 | 8.3 (5.9-12.2) | 10.3 (6.8-14.6) | 8.6 (5.9-12.9) | 9.8 (6.7-13.6) | 9.4 (6.0-13.0) | 9.4 (6.5-13.6) | 9.3 (6.4-13.3) | 8.7 (6.3-11.5) |
| RTE ($10^1$ U/$\mu$L) | 6.7 (3.6-12.0) | 4.5 (2.1-8.3) | 5.3 (2.5-10.1) | 5.5 (3.2-10.6) | 6.9 (3.4-12.4) | 5.1 (2.7-9.7) | 5.5 (2.9-10.5) | 20.7 (12.7-32.9) |
| RTE % CD4 | 20.9 (14.2-28.3) | 17.4 (9.9-23.8) | 18.4 (12.2-26.1) | 20.2 (12.1-28.9) | 19.0 (13.0-27.4) | 19.1 (12.9-26.5) | 19.0 (12.3-26.7) | 24.4 (17.1-33.2) |
| CD8 ($10^2$ U/$\mu$L) | 1.6 (1.1-2.5) | 1.3 (0.7-2.2) | 1.4 (0.9-2.4) | 1.4 (0.9-2.1) | 1.8 (1.1-2.8) | 1.3 (0.8-2.1) | 1.5 (0.9-2.4) | 4.9 (3.5-6.7) |
| CD8 % Lymph | 21.0 (14.0-26.0) | 18.0 (13.0-27.0) | 20.0 (14.0-26.0) | 19.0 (13.0-25.0) | 21.0 (14.5-27.5) | 19.0 (13.0-25.0) | 20.0 (14.0-26.0) | 25.0 (20.0-31.0) |
| HLA$^+$ % CD8 | 23.1 (14.3-33.3) | 31.0 (19.2-44.4) | 25.0 (15.4-36.3) | 27.3 (16.1-40.0) | 25.0 (17.1-36.8) | 26.3 (16.6-38.1) | 26.3 (16.7-38.5) | 23.8 (16.1-35.6) |
| CD19 ($10^1$ U/$\mu$L) | 11.5 (7.5-18.4) | 7.2 (4.3-11.6) | 9.2 (5.4-14.4) | 10.7 (6.3-15.7) | 9.9 (5.5-15.3) | 9.9 (5.9-15.2) | 9.8 (5.5-14.7) | 18.8 (13.0-26.3) |
| CD19 % Lymph | 15.0 (10.0-20.0) | 11.0 (7.0-17.0) | 12.0 (8.0-18.0) | 15.0 (11.0-21.0) | 11.0 (7.0-17.0) | 15.0 (10.0-20.5) | 13.0 (9.0-19.0) | 9.4 (7.0-12.0) |
| NK ($10^2$ U/$\mu$L) | 1.3 (0.8-2.0) | 1.2 (0.7-2.0) | 1.3 (0.8-2.1) | 1.1 (0.6-1.8) | 1.5 (0.8-2.1) | 1.2 (0.7-1.8) | 1.3 (0.7-2.0) | 3.0 (2.0-4.4) |
| NK % Lymph | 15.0 (10.0-23.0) | 18.4 (11.0-28.0) | 18.0 (12.0-27.0) | 15.0 (9.0-23.0) | 16.0 (10.0-24.0) | 16.0 (10.0-26.0) | 17.0 (10.0-25.0) | 15.0 (11.0-22.0) |

| cytokines | age < 70 | age ≥ 70 | $\Delta t_{ons} \leq 10$ | $\Delta t_{ons} > 10$ | WHO ≤ 2 | WHO > 2 | all inpatients | outpatients |
|---|---|---|---|---|---|---|---|---|
| IFN-$\gamma$ (pg/mL) | 1.7 (0.4-5.4) | 1.6 (0.3-4.5) | 2.1 (0.5-5.7) | 1.4 (0.2-4.3) | 1.1 (0.2-4.2) | 1.9 (0.5-5.1) | 1.7 (0.3-5.0) | - |
| IL10 ($10^1$ pg/mL) | 1.2 (0.7-2.2) | 1.5 (0.8-2.2) | 1.6 (0.9-2.3) | 1.1 (0.7-2.0) | 0.9 (0.6-1.6) | 1.7 (0.9-2.5) | 1.4 (0.7-2.2) | - |
| IL1B (pg/mL) | 7.7 (0.9-16.6) | 10.7 (1.6-18.8) | 10.9 (3.9-21.5) | 7.5 (0.3-15.4) | 6.3 (0.3-10.6) | 9.8 (0.3-20.2) | 8.5 (0.9-18.5) | - |
| IL2R ($10^3$ pg/mL) | 2.9 (2.2-3.9) | 3.5 (2.6-4.5) | 3.0 (2.3-4.0) | 3.4 (2.6-4.5) | 2.9 (2.1-4.1) | 3.4 (2.6-4.5) | 3.2 (2.3-4.2) | - |
| IL6 ($10^1$ pg/mL) | 2.9 (1.2-5.8) | 3.3 (1.9-7.2) | 3.4 (1.7-6.7) | 2.7 (1.2-5.4) | 2.1 (0.9-4.3) | 3.6 (1.9-8.2) | 3.0 (1.4-6.3) | - |
| IL8 ($10^1$ pg/mL) | 3.3 (2.2-5.2) | 4.1 (3.0-6.2) | 3.8 (2.6-5.9) | 3.4 (2.3-5.1) | 3.1 (2.0-4.4) | 4.2 (2.7-6.3) | 3.6 (2.6-5.7) | - |
| IP10 ($10^3$ pg/mL) | 1.1 (0.6-1.7) | 1.4 (1.0-1.8) | 1.4 (0.9-2.0) | 1.1 (0.6-1.6) | 0.9 (0.4-1.4) | 1.5 (1.0-2.0) | 1.2 (0.7-1.8) | - |

| biomarkers | age < 70 | age ≥ 70 | $\Delta t_{ons} \leq 10$ | $\Delta t_{ons} > 10$ | WHO ≤ 2 | WHO > 2 | all inpatients | outpatients |
|---|---|---|---|---|---|---|---|---|
| proADM (nmol/L) | 0.8 (0.7-1.1) | 1.2 (1.0-1.6) | 1.0 (0.8-1.4) | 0.9 (0.7-1.2) | 0.9 (0.7-1.3) | 1.1 (0.8-1.4) | 1.0 (0.8-1.3) | - |
| LDH ($10^2$ U/L) | 5.8 (4.4-7.5) | 6.2 (4.5-7.6) | 5.8 (4.4-7.3) | 6.6 (5.1-7.9) | 4.8 (3.8-6.4) | 6.8 (5.4-8.4) | 6.0 (4.5-7.6) | - |
| CRP ($10^1$ mg/L) | 6.9 (3.1-10.5) | 7.0 (3.4-11.3) | 7.1 (3.4-11.0) | 7.0 (3.7-11.3) | 4.7 (1.9-9.9) | 8.1 (4.5-12.2) | 7.0 (3.3-11.0) | - |

| outcomes | age < 70 | age ≥ 70 | $\Delta t_{ons} \leq 10$ | $\Delta t_{ons} > 10$ | WHO ≤ 2 | WHO > 2 | all inpatients | outpatients |
|---|---|---|---|---|---|---|---|---|
| infectious complications | 42/433 (10%) | 43/356 (12%) | 58/441 (13%) | 17/262 (6%) | 20/263 (8%) | 57/451 (13%) | 85/789 (11%) | - |
| OTI | 60/430 (14%) | 59/355 (17%) | 70/438 (16%) | 42/262 (16%) | 1/262 (0%) | 105/448 (23%) | 119/785 (15%) | - |
| death | 39/433 (9%) | 99/357 (28%) | 83/441 (19%) | 35/263 (13%) | 13/263 (5%) | 114/452 (25%) | 138/790 (17%) | - |
| death+OTI | 72/433 (17%) | 118/357 (33%) | 113/441 (26%) | 52/263 (20%) | 13/263 (5%) | 157/452 (35%) | 190/790 (24%) | - |
